# Epidemiology and risk factors of 28-day mortality of hospital-acquired bloodstream infection in Turkish intensive care units: a prospective observational cohort study

**DOI:** 10.1093/jac/dkad167

**Published:** 2023-06-02

**Authors:** Abdullah Tarık Aslan, Alexis Tabah, Bahadır Köylü, Ayşe Kaya Kalem, Firdevs Aksoy, Çiğdem Erol, Rıdvan Karaali, Burcu Tunay, Seda Guzeldağ, Ayşe Batirel, Emine Kübra Dindar, Özlem Akdoğan, Yeliz Bilir, Gülden Ersöz, Barçın Öztürk, Mehtap Selçuk, Mesut Yilmaz, Ahmet Akyol, Türkay Akbaş, Hülya Sungurtekin, Arif Timuroğlu, Yunus Gürbüz, Onur Çolak, Yaşar Bayindir, Ahmet Eroğlu, Leyla Ferlicolak, Utku Çeşme, Osman Dağ, Niccoló Buetti, François Barbier, Stéphane Ruckly, Quentin Staiquly, Jean-François Timsit, Murat Akova

**Affiliations:** Hacettepe University, Faculty of Medicine, Department of Internal Medicine, Ankara, Turkey; The University of Queensland Centre for Clinical Research, Faculty of Medicine, The University of Queensland, Brisbane, QLD 4029, Australia; The University of Queensland Centre for Clinical Research, Faculty of Medicine, The University of Queensland, Brisbane, QLD 4029, Australia; Intensive Care Unit, Redcliffe Hospital, Metro North Hospital and Health services, Queensland, Australia; Faculty of Medicine, The University of Queensland, Brisbane, Queensland; Hacettepe University, Faculty of Medicine, Department of Internal Medicine, Ankara, Turkey; Department of Infectious Diseases and Clinical Microbiology, Ankara City Hospital, Ankara, Turkey; Department of Infectious Diseases and Clinical Microbiology, Karadeniz Technical University, School of Medicine, Trabzon, Turkey; Department of Infectious Diseases and Clinical Microbiology, Baskent University, Ankara, Turkey; Department of Infectious Diseases and Clinical Microbiology, Faculty of Medicine, Istanbul University, Istanbul, Turkey; Department of Anesthesiology and Reanimation, Istanbul Medipol University, Medipol Mega University Hospitals, Istanbul, Turkey; Department of Internal Medicine, Division of Intensive Care Medicine, Kayseri City Hospital, Kayseri, Turkey; Department of Infectious Diseases and Clinical Microbiology, University of Health Sciences Kartal Dr. Lutfi Kırdar Training and Research Hospital, Istanbul, Turkey; Department of Infectious Diseases and Clinical Microbiology, Bitlis State Hospital, Bitlis, Turkey; Department of Infectious Diseases and Clinical Microbiology, Erol Olçok Education and Research Hospital, Hitit University, Çorum, Turkey; Department of Anesteshiology and Reanimation, Division of Intensive Care Medicine, Kartal Dr. Lutfi Kırdar Training and Research Hospital, Istanbul, Turkey; Department of Infectious Diseases and Clinical Microbiology, Mersin University Hospital, Mersin, Turkey; Department of Infectious Diseases and Clinical Microbiology, Faculty of Medicine, Aydın Adnan Menderes University, Aydın, Turkey; Department of Internal Medicine, Division of Intensive Care Medicine, Acibadem Kadıköy Hospital, Istanbul, Turkey; Department of Infectious Diseases and Clinical Microbiology, Istanbul Medipol University, Koşuyolu Medipol Hospital, Istanbul, Turkey; Department of Anesteshiology and Reanimation, Division of Intensive Care Medicine, Health Science University, Ümraniye Training and Research Hospital, Istanbul, Turkey; Department of Internal Medicine, Division of Intensive Care Medicine, Faculty of Medicine, Düzce University, Düzce, Turkey; Department of Internal Medicine, Division of Intensive Care Medicine, Pamukkale University Hospital, Denizli, Turkey; Department of Internal Medicine, Division of Intensive Care Medicine, SBU Dr. AY Ankara Oncology Research and Training Hospital, Ankara, Turkey; Department of Infectious Diseases and Clinical Microbiology, University of Health Sciences, Dışkapı Yıldırım Beyazıt Hospital, Ankara, Turkey; Department of Infectious Diseases and Clinical Microbiology, Fatih Sultan Mehmet Research and Training Hospital, Istanbul, Turkey; Department of Infectious Diseases and Clinical Microbiology, Turgut Özal Medical Center, Malatya, Turkey; Department of Anesteshiology and Reanimation, Division of Intensive Care Medicine, Karadeniz Technical University, Farabi Hospital, Trabzon, Turkey; Department of Internal Medicine, Division of Intensive Care Medicine, Ibni Sina Hospital, Ankara University, Ankara, Turkey; Faculty of Medicine, Hacettepe University, Ankara, Turkey; Faculty of Medicine, Department of Biostatistics, Hacettepe University, Ankara, Turkey; Infection Control Programme, University of Geneva Hospitals and Faculty of Medicine, WHO Collaborating Center, Geneva, Switzerland; Unité Mixte de Recherche (UMR) 1137, IAME, Université Paris Cité, 75018 Paris, France; Hopital de la Source, Intensive Care Unit, Orléans, France; Research, Outcomerea and ICUREsearch, Paris, France; Research, Outcomerea and ICUREsearch, Paris, France; Bichat Hospital, Medical and Infectious Diseases Intensive Care Unit, Paris, France; Faculty of Medicine, Infectious Diseases and Clinical Microbiology, Hacettepe University, Ankara, Turkey

## Abstract

**Objectives:**

To uncover clinical epidemiology, microbiological characteristics and outcome determinants of hospital-acquired bloodstream infections (HA-BSIs) in Turkish ICU patients.

**Methods:**

The EUROBACT II was a prospective observational multicontinental cohort study. We performed a subanalysis of patients from 24 Turkish ICUs included in this study. Risk factors for mortality were identified using multivariable Cox frailty models.

**Results:**

Of 547 patients, 58.7% were male with a median [IQR] age of 68 [55–78]. Most frequent sources of HA-BSIs were intravascular catheter [182, (33.3%)] and lower respiratory tract [175, (32.0%)]. Among isolated pathogens (*n* = 599), 67.1% were Gram-negative, 21.5% Gram-positive and 11.2% due to fungi. Carbapenem resistance was present in 90.4% of *Acinetobacter* spp., 53.1% of *Klebsiella* spp. and 48.8% of *Pseudomonas* spp. In monobacterial Gram-negative HA-BSIs (*n* = 329), SOFA score (aHR 1.20, 95% CI 1.14–1.27), carbapenem resistance (aHR 2.46, 95% CI 1.58–3.84), previous myocardial infarction (aHR 1.86, 95% CI 1.12–3.08), COVID-19 admission diagnosis (aHR 2.95, 95% CI 1.25–6.95) and not achieving source control (aHR 2.02, 95% CI 1.15–3.54) were associated with mortality. However, availability of clinical pharmacists (aHR 0.23, 95% CI 0.06–0.90) and source control (aHR 0.46, 95% CI 0.28–0.77) were associated with survival. In monobacterial Gram-positive HA-BSIs (*n* = 93), SOFA score (aHR 1.29, 95% CI 1.17–1.43) and age (aHR 1.05, 95% CI 1.03–1.08) were associated with mortality, whereas source control (aHR 0.41, 95% CI 0.20–0.87) was associated with survival.

**Conclusions:**

Considering high antimicrobial resistance rate, importance of source control and availability of clinical pharmacists, a multifaceted management programme should be adopted in Turkish ICUs.

## Introduction

Hospital-acquired bloodstream infection (HA-BSI) is one of the leading causes of severe sepsis and septic shock, with a high risk of morbidity, mortality and treatment costs.^1–4^ Overall, 5%–7% of patients develop ICU-acquired BSI during the ICU stay, which corresponds to an average of 6–10 cases per 1000 patient-days.^2,5^

Over the last two decades, the alarmingly increasing prevalence of antimicrobial-resistant organisms has led to the more frequent use of broad-spectrum antibiotics, creating a vicious cycle that facilitates the emergence of antimicrobial-resistant organisms.^6^ Therefore, several organizations and agencies have issued a number of publications alerting people to this growing public health threat.^6,7^

The EUROBACT II study was a prospective observational multinational cohort study (ClinicalTrials.org registration ID NCT03937245).^8^ More than one-fifth of the patients included in the EUROBACT II study were from Turkish ICUs. Because the epidemiology and antimicrobial resistance profile of Turkey is different from that of other countries (e.g. *Acinetobacter* spp.-related bloodstream infections are common, carbapenem resistance rate is very high among Gram-negatives), a separate analysis was planned for the cohort of Turkish patients in the preparation phase of the EUROBACT II study. Despite its clinical importance, little is known about the clinical epidemiology, microbiological characteristics and outcome determinants of HA-BSIs in Turkish ICU patients. Considering the high frequencies of antimicrobial-resistant Gram-negative bacterial (GNB) infections in Turkey, we aimed to investigate the risk factors of mortality and the characteristics of causative pathogens in critically ill patients with HA-BSI in this pre-planned analysis. Data from this study can be used to improve patient care, monitor trends, develop local guidelines and serve as a benchmark for future studies.

## Methods

In this study, an analysis of patients enrolled in the EUROBACT II study from Turkish ICUs was conducted and reported in accordance with the STrengthening the Reporting of OBservational studies in Epidemiology (STROBE) guidelines.^9^

### Ethics

This study was approved by the Hacettepe University Faculty of Medicine Clinical Research Ethics Committee (ref no. KA-19126), and informed consent was obtained from all patients and/or their family members.

### ICU and patient recruitment

All consecutive adult patients (≥18 years of age) with an initial episode of HA-BSI treated in the ICU were enrolled between 24 August 2019 and 21 February 2021. The definitions of ICU and HA-BSI have been described previously.^8^ Patients with blood culture positivity with a typical skin contaminant were included if the same pattern of antimicrobial susceptibility profile was identified by at least two consecutive blood culture samples and/or strong clinical grounds (e.g. infected catheter proven to be a source for HA-BSI) existed.^8^ In the case of recurrent HA-BSIs, only the first episode was included for the analysis. Inclusion and exclusion criteria were described in the EUROBACT II protocol.^8^

Case recruitment continued in this study for at least 3 months or until 10 consecutive HA-BSIs treated in the ICU were included (whichever came first), and centres that volunteered were able to continue to include cases throughout the entire study period. To facilitate participation in this study, each ICU was allowed to enrol at least 10 cases in any 3 months of the patient enrolment period.

### Data collection

Data were obtained from the hospital registries and patient files without performing any additional interventions. Relevant clinical and microbiological data were collected, along with those related to ICU and microbiology laboratory characteristics. Severity of illness was interpreted by the Simplified Acute Physiology Score II (SAPS II), and the SOFA score at ICU admission and HA-BSI diagnosis, respectively.^10,11^ Sepsis and septic shock were defined according to Sepsis III criteria.^12^ Blood culture sampling time was accepted as the time zero of the HA-BSI episode from which all timings were calculated (e.g. time to appropriate antimicrobial therapy). Sources of HA-BSI were assessed by the attending physicians and ordered according to clinical probability. If an HA-BSI episode had no identified origin, it was defined as primary HA-BSI. All recruited patients were followed for 28 days or death. Data on antimicrobial therapies were collected starting 2 days before HA-BSI until ICU discharge or the end of follow-up on day 28. Appropriate therapy was defined as receiving at least one *in vitro* active regimen with an adequate dosing for the microorganism within 5 days of blood culture sampling and was assessed by three members of the steering committee (N.B., A.T. and F.B.). Time to appropriate antimicrobial therapy was defined as the time between culture sampling of the first HA-BSI episode and receipt of at least one *in vitro* active antimicrobial therapy for each microorganism. Carbapenem-resistant *Enterobacterales* was defined according to the US Centers for Disease Control and Prevention criteria.^13^ Difficult-to-treat resistance (DTR) in GNB was determined according to the criteria defined by Kadri *et al*.^14^ Pan-drug-resistance (PDR) was described as resistance to all tested antimicrobials. DTR and PDR were assessed for *Enterobacterales*, *Pseudomonas* spp. and *Acinetobacter* spp. To avoid over-estimating DTR and PDR for microorganisms with inadequately reported antibiograms, the interpretation required availability of susceptibility results for at least one fluoroquinolone, one cephalosporin, one carbapenem and plus polymyxins for PDR. Most antimicrobial susceptibility results were interpreted according to EUCAST guidelines and 10% of them were reported based on CLSI recommendations.^15,16^ Adequacy of source control was assessed by the local investigators. At least two different infectious diseases or intensive care specialists from each centre took part in determining the adequacy of source control. At least one of these investigators from each centre has more than 10 years of clinical experience. Any disagreement in assessing the adequacy of source control was resolved through discussion. The time, date and effectiveness of the intervention were recorded according to predefined categories. When patients had multiple source control interventions, the number of interventions and the date of the last intervention were also recorded. Further information on definitions is presented in the original report.^8^

### Statistical analyses

A dual verification and inquiry process was used, which included automatic validation of all data collected through a set of consistency routines and manual checking of each case report form by the principal investigators for data quality and completeness. Queries that emerged after the data review were sent to local researchers through the electronic case report form-embedded system and checked multiple times until these queries were sufficiently resolved.^8^ Continuous variables were presented as medians (IQR) and categorical variables as absolute frequencies and percentages. Comparisons were performed by Wilcoxon rank-sum test for continuous variables and Pearson’s chi-squared test or Fisher’s exact test for categorical variables.

All variables not correlated and involved in more than 5% of the cohort with a *P* value < 0.10 by univariate analysis and associated with 28-day mortality were incorporated into the multivariable Cox frailty models. A random effect for centre was included. Because of the presence of a small number of missing values, simple imputation to the mode for categorical variables was used. Survival curves were obtained using the Kaplan–Meier method and compared using the log rank test. A sensitivity analysis of Gram-negative monobacterial HA-BSIs subgroup was also undertaken by excluding the COVID-19 patients. A subgroup analysis of monobacterial Gram-negative HA-BSIs was performed in patients treated with appropriate antimicrobial therapy to analyse the effect of appropriate therapy initiated within 24 hours of blood culture sampling on 28-day mortality. Statistical analysis was done using SPSS v.26.0 (IBM Corp., Armonk, NY, USA) and R v.4.0.2. In all analyses, two-sided *P* values less than 0.05 were deemed statistically significant.

## Results

### Main characteristics of centres and patients

In this study, 547 patients from 24 Turkish ICUs were included (Figure S1, available as Supplementary data at *JAC* Online). Four centres were excluded from the study with following reasons: no patients included (*n* = 1), closure documents not provided prior to database lockdown (*n* = 1) and poor quality of data (*n* = 2). Additionally, 32 patients were excluded for various reasons, as depicted in Figure S1. The characteristics of recruited ICUs are presented in Table 1 and Table S1. Most participating ICUs were mixed (medical-surgical) type ICUs located in a public teaching hospital. Therapeutic drug monitoring for aminoglycosides, vancomycin and β-lactams, as well as availability of clinical pharmacists were quite seldom in participating ICUs (Table 1). Only 25% of the hospitals declared to have local infection treatment guidelines and 12.5% had either molecular or phenotypic rapid diagnostic tests for identification of resistance mechanisms.

**Table 1.** Characteristics of participating ICUs according to survival status at day-28

Characteristics	All ICUs (*n* = 24)	All patients (*N* = 547)	Non-survivors (*n* = 269)	Survivors (*n* = 278)	*P* value
Academic status of the hospital					<0.001
Teaching hospital	22 (91.7)	500 (91.4)	259 (96.3)	241 (86.7)	
Non-teaching hospital	2 (8.3)	47 (8.6)	10 (3.7)	37 (13.3)	
Type of ICU					0.008
Mixed (medical-surgical)	20 (83.3)	404 (73.9)	185 (68.8)	219 (78.8)	
Medical	4 (16.7)	143 (26.1)	84 (31.2)	59 (21.2)	
Number of ventilator equivalent beds in the ICU < 15	13 (54.2)	305 (55.7)	124 (46.1)	181 (65.1)	0.084
Nurse to ventilator-bed ratio	2.2 [1.7–2.5]	2.3 [1.8–2.6]	2.3 [1.8–2.6]	2.3 [1.8–3.0]	0.196
Senior doctor to ventilator-bed ratio	7 [5.4–10.1]	7 [5.3–11.0]	7 [5.3–11.0]	7 [5.3–11.0]	0.961
Senior medical cover is available 24/7	19 (79.2)	435 (79.5)	222 (82.5)	213 (76.6)	0.087
General surgery is available 24/7	24 (100.0)	547 (100.0)	269 (100.0)	278 (100.0)	NC
Infectious diseases specialist or clinical microbiologist are consulted					0.393
Available when requested 24/7	20 (87.0)	457 (85.1)	222 (83.8)	235 (86.4)	
As a permanent staff of the ICU	3 (13.0)	80 (14.9)	43 (16.2)	37 (13.6)	
Clinical pharmacists are consulted					0.038
Available when requested 24/7	3 (13.0)	58 (11.2)	21 (8.1)	37 (14.3)	
Never or sporadically	20 (87.0)	459 (88.8)	237 (91.9)	222 (85.7)	
TDM of aminoglycosides is available					0.279
Everyday	1 (4.2)	10 (1.8)	3 (1.1)	7 (2.5)	
At least once a week	3 (12.5)	45 (8.2)	19 (7.1)	26 (9.4)	
Not available	20 (80.3)	492 (89.9)	247 (91.8)	245 (88.1)	
TDM of vancomycin is available					0.049
Everyday	2 (8.3)	37 (6.8)	24 (8.9)	13 (4.7)	
At least once a week	4 (16.7)	52 (9.5)	20 (7.4)	32 (11.5)	
Not available	18 (75.0)	458 (83.7)	225 (83.6)	233 (83.8)	
TDM of β-lactams is available					0.065
Everyday	1 (4.2)	10 (1.8)	3 (1.1)	7 (2.5)	
At least once a week	2 (8.3)	16 (2.9)	4 (1.5)	12 (4.3)	
Not available	21 (87.5)	521 (95.2)	262 (97.4)	259 (93.2)	

Results are presented as *n* (%) for categorical variables and median [IQR] for continuous variables. Ventilator equivalent denotes the maximum number of ventilated patients the ICU can accommodate at one time. 24/7, 24 hours a day, 7 days a week; ICU, intensive care unit; TDM, therapeutic drug monitoring

Of 547 patients, 58.7% were male with a median [IQR] age of 68 [55–78] and 81.5% had at least one underlying comorbidity. The most frequent primary ICU admission diagnosis was COVID-19 (26.0%), followed by non-COVID-19-related respiratory diseases (24.9%) and neurologic causes (15.7%). Co-morbidities and ICU admission diagnosis are shown in Table S2. Among all patients, the interval between hospital admission and drawing of positive blood culture was 14 [8–29] days, with 12.2% sampled before ICU admission, 24.3% within the first week following ICU admission, and 63.4% after this timepoint. At the onset of HA-BSI, the median SOFA score was 8 [6–11], with 71.6% and 25.7% were presented with sepsis and septic shock, respectively (Table 2). Main sources of HA-BSI were intravascular catheter (33.3%) and lower respiratory tract (32.0%), followed by primary HA-BSI (17.7%), while 17.4% of the patients had multiple possible sources.

**Table 2.** Baseline characteristics of the patients according the mortality status at day 28

Variable	All patients (*n* = 547)	Non-survivors (*n* = 269)	Survivors (*n* = 278)	*P* value
Patient characteristics on ICU admission				
Age (years)	68 [55–78]	71 [59–79]	65 [50–76]	<0.001
SAPS II score	49 [38–61]	49 [39–63]	49 [37–59]	0.149
Male gender	321 (58.7)	164 (61.0)	157 (56.5)	0.352
Body mass index (kg/m^2^)				0.524
<18.5	11 (2.0)	4 (1.5)	7 (2.5)	
18.5–30.0	410 (74.9)	202 (75.1)	208 (74.8)	
≥30	126 (23.1)	63 (23.4)	63 (22.7)	
Charlson comorbidity index				0.008
0	108 (19.7)	39 (14.5)	69 (24.8)	
1–2	240 (43.9)	129 (48.0)	111 (39.9)	
>2	199 (36.4)	101 (37.5)	98 (35.3)	
Solid tumour, no metastasis	47 (8.6)	30 (11.2)	17 (6.1)	0.046
Solid tumour, with metastasis	52 (9.5)	26 (9.7)	26 (9.4)	0.901
Haematological malignancy	25 (4.6)	16 (5.9)	9 (3.2)	0.189
Type of ICU admission				0.023
Medical	479 (87.6)	246 (91.4)	233 (83.8)	
Surgical elective	21 (3.8)	8 (3.0)	13 (4.7)	
Surgical emergency	47 (8.6)	15 (5.6)	32 (11.5)	
Primary ICU admission diagnosis				<0.001
Sepsis or septic shock	48 (8.8)	24 (8.9)	24 (8.6)	
Respiratory admission^a^	136 (24.9)	68 (25.3)	68 (24.5)	
COVID-19^a^	142 (26.0)	104 (38.7)	38 (13.7)	
Post-operative admission	27 (4.9)	10 (3.7)	17 (6.1)	
Other admission diagnoses	194 (35.4)	63 (23.4)	131 (47.1)	
Patient characteristics at HA-BSI diagnosis				
Time from ICU admission to HA-BSI				0.005
Late ICU-acquired (>7 days)	347 (63.4)	160 (59.5)	187 (67.3)	
Acquired prior to ICU admission	67 (12.2)	26 (9.7)	41 (14.7)	
Early ICU-acquired (≤7 days)	133 (24.3)	83 (30.9)	50 (18.0)	
Maximum temperature				0.010
<38.2°C	388 (71.1)	204 (76.1)	184 (66.2)	
≥38.2°C	158 (28.9)	64 (23.9)	94 (33.8)	
Sepsis or septic shock				<0.001
No sepsis or sepsis without shock	405 (74.3)	175 (65.5)	230 (82.7)	
Septic shock—no steroids	91 (16.7)	52 (19.5)	39 (14.0)	
Septic shock—steroids administered	49 (9.0)	40 (15.0)	9 (3.2)	
SOFA score	8 [6–11]	9 [6–12]	7 [5–9]	<0.001
Ventilation status				<0.001
Low flow oxygen or no oxygen	82 (15.0)	24 (8.9)	58 (20.9)	
High flow oxygen nasal canula	34 (6.2)	14 (5.2)	20 (7.2)	
Non-invasive mechanical ventilation or CPAP	39 (7.1)	14 (5.2)	25 (9.0)	
Invasive mechanical ventilation	392 (71.7)	217 (80.7)	175 (62.9)	
Vasopressors (adrenaline or noradrenaline)	221 (40.4)	136 (50.6)	85 (30.6)	<0.001
Gram-negative bacteria^b^	384 (70.2)	189 (70.3)	195 (70.1)	0.859
DTR Gram-negative	135 (24.7)	85 (31.6)	50 (18.0)	0.012
Carbapenem-resistant Gram-negative	209 (38.2)	130 (48.3)	79 (28.4)	<0.001
Gram-positive bacteria^b^	123 (22.5)	71 (26.4)	52 (18.7)	0.05
Resistant Gram-positive (MRSA, MRCNS or VRE)	47 (8.6)	30 (11.2)	17 (6.1)	0.069
Fungus^b^	67 (12.2)	30 (11.2)	37 (13.3)	0.356
Strict anaerobe bacteria^b^	1 (0.2)	0 (0)	1 (0.4)	
Polymicrobial blood culture	48 (8.8)	28 (10.4)	20 (7.2)	0.186
Source of HA-BSI				0.461
Intravascular catheter	182 (33.3)	92 (34.2)	90 (32.4)	
Intra-abdominal	28 (5.1)	11 (4.1)	17 (6.1)	
Primary	97 (17.7)	47(17.5)	50 (18.0)	
Respiratory	175 (32.0)	92 (34.2)	83 (29.9)	
Urinary	42 (7.7)	18 (6.7)	24 (8.6)	
Other	23 (4.2)	9 (3.3)	14 (5.0)	
More than one possible source of infection	95 (17.4)	43 (16.0)	52 (18.7)	0.779
Time to *in vitro* active antimicrobial therapy				0.008
≤24 hours, *n* (%)	233 (42.6)	118 (43.9)	115 (41.3)	
24–48 hours, *n* (%)	69 (12.6)	31 (11.5)	38 (13.7)	
48–120 hours, *n* (%)	94 (17.2)	34 (12.6)	60 (21.6)	
>120 hours, *n* (%)	42 (7.7)	15 (5.6)	27 (9.7)	
Never, *n* (%)	109 (19.9)	71 (26.4)	38 (13.7)	
Source control				<0.001
Not required	323 (59.0)	161 (59.9)	162 (58.2)	
Required, achieved	165 (30.2)	62 (23.0)	103 (37.1)	
Required, but NOT achieved	59 (10.8)	46 (17.1)	13 (4.7)	

Respiratory admission refers to admission for respiratory failure other than COVID-19.

Sum of percentages exceeds 100 because a patient may have had a polymicrobial HA-BSI. SAPS II, Simplified Acute Physiology Score II; CPAP, continuous positive airway pressure; MRCNS, methicillin-resistant coagulase-negative *Staphylococci*.

Continuous variables are presented as median [IQR]. Categorical variables are presented as *n* (%). Closed brackets indicate inclusive of the end of the range and open brackets indicate the exclusion of the end of the range.

### Features of microorganisms

Among all blood culture samples including bacterial and fungal isolates (*n* = 599), 91.2% and 8.8% were monomicrobial and polymicrobial, respectively. Pathogens were predominantly Gram-negative (402/599; 67.1%): *Acinetobacter* spp. (135/402; 33.6%), *Klebsiella* spp. (113/402; 28.1%), *Escherichia coli* (49/402; 12.2%) and *Pseudomonas* spp. (41/402; 10.2%), by order of pathogen count (Table 3). Carbapenem resistance was detected in 90.4% (122/135) of *Acinetobacter* spp., 53.1% (60/113) of *Klebsiella* spp., 48.8% (20/41) of *Pseudomonas* spp., 33.3% (8/24) of *Enterobacter* spp. and 6.1% (3/49) of *E. coli*. Similarly, DTR was present in 57.8% (78/135) of *Acinetobacter* spp., 39.8% (45/113) of *Klebsiella* spp., 19.5% (8/41) of *Pseudomonas* spp. and 16.7% (4/24) of *Enterobacter* spp. Gram-positive bacteria (129/599; 21.5%) mainly composed of *Enterococcus* spp. (55/129, 42.6%) and *Staphylococcus aureus* (35/129, 27.1%). Among *S. aureus* isolates, 42.9% (15/35) were MRSA. Intriguingly, methicillin resistance rate was very high among coagulase-negative *staphylococci* (25/32, 78.1%). There was only one strict anaerobe bacteria. 11.2% (67/599) of the pathogens were fungi and 65.7% (44/67) of them were *non-albicans Candida* spp. and 34.3% (23/67) were *Candida albicans*.

**Table 3.** Phenotypic characteristics of the pathogens

Pathogens (*n* = 599)^a^	Results (*n*, %)
Gram-negative bacteria	402 (67.1)
*Klebsiella* spp.	113 (28.1)
Carbapenem resistant	60 (53.1)
DTR	45 (39.8)
PDR	9 (8.0)
*Escherichia coli*	49 (12.2)
Carbapenem resistant	3 (6.1)
DTR	0 (0.0)
PDR	0 (0.0)
*Enterobacter* spp.	24 (6.0)
Carbapenem resistant	8 (33.3)
DTR	4 (16.7)
PDR	0 (0.0)
*Pseudomonas* spp.	41 (10.2)
Carbapenem resistant	20 (48.8)
DTR	8 (19.5)
PDR	2 (4.9)
*Acinetobacter* spp.	135 (33.6)
Carbapenem resistant	122 (90.4)
DTR	78 (57.8)
PDR	3 (2.2)
Other Gram-negative bacteria	40 (10.0)
Carbapenem resistant^b^	1 (4.5)
Gram-positive bacteria	129 (21.5)
*Enterococcus* spp.	55 (42.6)
*Enterococcus faecium*	30 (54.5)
VRE	8 (26.7)
Coagulase-negative *Staphylococcus*	32 (24.8)
MRCNS	25 (78.1)
*Staphylococcus aureus*	35 (27.1)
MRSA	15 (42.9)
Other Gram-positive bacteria	7 (5.4)
Strict anaerobe bacteria	1 (0.2)
Fungi	67 (11.2)
*Candida albicans*	23 (34.3)
*Non-albicans Candida* spp.	44 (65.7)

In this study, 599 pathogens were isolated from 547 patients.

The carbapenem resistance rate of other Gram-negative bacteria after excluding *Stenotrophomonas maltophilia* isolates (*n* = 18) is shown. If the species of Candida was not reported, they were classified as non-albicans.

PDR, pan-drug resistant; GNB, Gram-negative bacteria; MRCNS, methicillin-resistant coagulase-negative *staphylococci*.

### Management of patients

**Figure 1.**
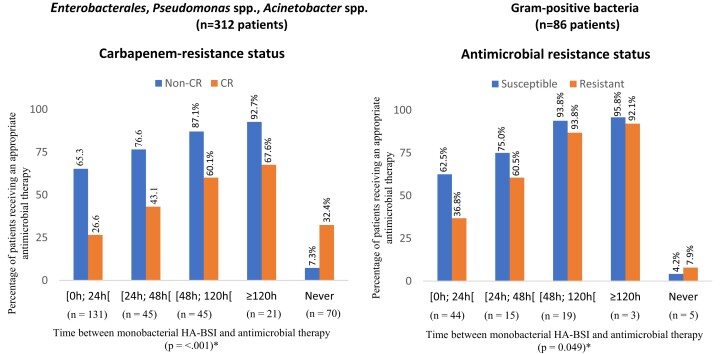
Relationships between antibiotic resistance and timing of appropriate antimicrobial therapy. Cumulative percentage of patients treated with at least one *in vitro* active antimicrobial, on each time period before and after the date of drawing of the initial positive blood culture, demonstrated by antimicrobial resistance status. In the first graph (located on the left-hand side), carbapenem resistance was interpreted among *Enterobacterales*, *Pseudomonas* spp. and *Acinetobacter* spp. In the second graph (located on the right-hand side), ‘resistant’ indicates the presence of methicillin resistance among coagulase-negative *staphylococci* spp. and *Staphylococcus aureus*, as well as the presence of vancomycin resistance in *Enterococci* spp. Closed brackets denote inclusive of the end of the range and open brackets denote the exclusion of the end of the range. *Chi-square test for linear-by-linear association was used for statistical purposes. Non-CR, non-carbapenem resistant; CR, carbapenem resistant. This figure appears in colour in the online version of *JAC* and in black and white in the print version of *JAC*.

**Figure 2.**
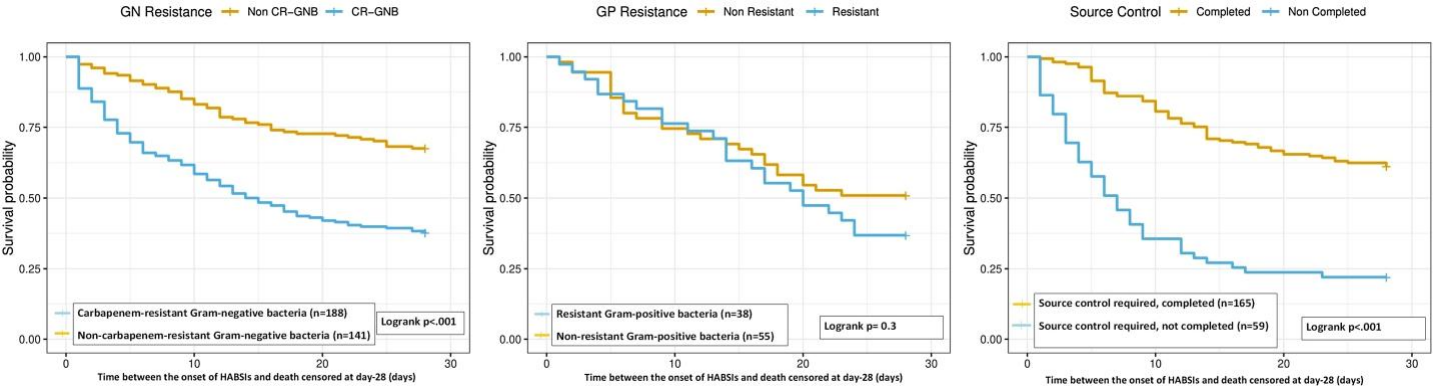
Survival curves for monobacterial Gram-negative HA-BSIs, monobacterial Gram-positive HA-BSIs and HA-BSIs requiring source control. In survival probability analysis of monobacterial Gram-negative HA-BSIs, infections caused by *Stenotrophomonas maltophilia* were excluded. In the middle figure for monobacterial Gram-positive HA-BSIs, ‘resistance’ indicates the presence of methicillin resistance among coagulase-negative *staphylococci* spp., and *Staphylococcus aureus*, as well as the presence of vancomycin resistance in *Enterococci* spp. In the survival probability analysis of source control, only patients requiring source control were considered. GN, Gram-negative; CR-GNB, carbapenem-resistant Gram-negative bacteria; GP, Gram-positive. This figure appears in colour in the online version of *JAC* and in black and white in the print version of *JAC*.

Appropriate antimicrobial therapies were administered for 42.6% and 55.2% of the patients within 24 and 48 hours of blood culture sampling, respectively. As shown in Figure 1, antimicrobial resistance had a detrimental effect on early prescription of at least one appropriate antimicrobial agent. All appropriate antimicrobial regimens are listed in Table S3. Source control was required in 41.0% of the patients and was successfully undertaken in 73.7% of these, after a median of 2 [1–5] days. Among patients requiring source control, it was primarily achieved by following interventions: removal of intravascular catheters (*n* = 124), debridement and/or drainage for bone and/or soft tissue infections (*n* = 10), surgical intervention for bone and/or soft tissue infections (*n* = 5), surgical or percutaneous interventions for intra-abdominal infections (*n* = 8), removal of urinary catheters (*n* = 9), urinary surgical procedure (*n* = 1), percutaneous interventions for respiratory tract infections including empyema (*n* = 4) and others including endoscopic source control interventions (*n* = 4). 49.1% (269/547) of the patients died by day-28, with most occurring during ICU stay (259/547; 47.3%). Of those who survived at that time, 62.9% of the survivors were still in the ICU, 15.1% continued to be treated in a non-ICU ward and only 21.9% were discharged from the hospital. The survival probabilities of three different comparison groups are shown in Figure 2.

### Factors associated with mortality in Gram-negative and Gram-positive monobacterial HA-BSIs

Baseline characteristics of patients with monobacterial Gram-negative and Gram-positive HA-BSI are presented in Tables S4 and S5 and Tables S6 and S7, respectively. Associations between risk factors and mortality for both Gram-negative and Gram-positive monobacterial infections are shown in Table 4. In monobacterial Gram-negative HA-BSIs, SOFA score (aHR 1.20, 95% CI 1.14–1.27), carbapenem resistance (aHR 2.46, 95% CI 1.58–3.84), previous myocardial infarction (aHR 1.86, 95% CI 1.12–3.08), COVID-19 admission diagnosis (aHR 2.95, 95% CI 1.25–6.95) and not achieving source control (aHR 2.02, 95% CI 1.15–3.54) were associated with mortality. However, availability of clinical pharmacists (aHR 0.23, 95% CI 0.06–0.90) and source control (aHR 0.46, 95% CI 0.28–0.77) were associated with survival. In monobacterial Gram-positive HA-BSIs, SOFA score (aHR 1.29, 95% CI 1.17–1.43) and age (aHR 1.05, 95% CI 1.03–1.08) were associated with mortality, whereas source control (aHR 0.41, 95% CI 0.20–0.87) was associated with survival. As COVID-19 was associated with higher risk of mortality in monobacterial HA-BSIs caused by GNB, a sensitivity analysis of GNB infections not including COVID-19 patients was performed (Tables S8–S10). In this analysis, carbapenem resistance (aHR 3.32, 95% CI 1.93–5.72), haematological malignancy (aHR 2.46, 95% CI 1.12–5.39), diabetes with end-organ damage (aHR 4.07, 95% CI, 1.92–8.67), respiratory disease as an admission diagnosis (aHR 2.91, 95% CI 1.26–6.71) and SOFA score (aHR 1.29, 95% CI 1.20–1.39) were independent predictors of 28-day mortality. However, availability of clinical pharmacists (aHR 0.06, 95% CI 0.01–0.41), and complete source control (aHR 0.38, 95% CI 0.19–0.74) were independent predictors of survival. In the subgroup analysis including appropriately treated monobacterial HA-BSIs caused by GNB, early appropriate therapy did not provide a significant survival benefit over appropriate therapies starting between 24 and 48 hours (aHR 0.86, 95% CI 0.47–1.55), and 48 and 120 hours (aHR 0.60, 95% CI 0.29–1.22).

**Table 4.** Multivariable Cox frailty regression analyses for both monobacterial Gram-negative and Gram-positive HA-BSIs

Gram-negative monobacterial HA-BSIs (*n* = 329)	Univariate analysis		Multivariate analysis	
Variable	HR [95% CI]	*P* value	aHR [95% CI]	*P* value
Medical ICU	1.66 [1.09–2.52]	0.018	1.36 [0.60–3.12]	0.463
Availability of clinical pharmacists when requested 24/7	0.48 [0.25–0.91]	0.023	0.23 [0.06–0.90]	0.035
Maximum temperature ≥38.2°C	0.64 [0.44–0.92]	0.016	0.67 [0.43–1.05]	0.080
SOFA score	1.19 [1.16–1.23]	<0.001	1.20 [1.14–1.27]	<0.001
Carbapenem resistance	3.42 [2.24–5.21]	<0.001	2.46 [1.58–3.84]	<0.001
Appropriate therapy	0.68 [0.48–0.95]	0.024	0.77 [0.51–1.16]	0.216
Heart failure (NYHA 3)	1.76 [1.13–2.75]	0.012	1.62 [0.91–2.89]	0.102
Previous myocardial infarction	1.57 [1.00–2.47]	0.051	1.86 [1.12–3.08]	0.016
Cerebrovascular disease	0.42 [0.24–0.74]	0.003	0.54 [0.27–1.06]	0.073
Charlson comorbidity index >2	1.17 [0.94–1.47]	0.095	1.03 [0.78–1.36]	0.849
TDM of β-lactams is available				
Not available	Ref.		Ref.	
At least once a week	0.50 [0.18–1.34]	0.099	1.18 [0.26–5.26]	0.830
Everyday	0.34 [0.08–1.37]	0.098	1.00 [0.09–10.94]	0.997
Type of ICU admission				
Medical	Ref.		Ref.	
Surgical elective	1.53 [0.62–3.70]	0.345	1.42 [0.52–3.88]	0.493
Surgical emergency	2.00 [0.93–4.34]	0.074	1.68 [0.67–4.22]	0.269
Primary ICU admission diagnosis				
Sepsis and septic shock	Ref.		Ref.	
Respiratory	1.14 [0.52–2.50]	0.735	1.79 [0.80–3.97]	0.154
COVID-19	2.34 [1.08–5.06]	0.030	2.95 [1.25–6.95]	0.013
Post-operative admission	0.42 [0.11–1.62]	0.209	0.26 [0.06–1.11]	0.069
Other admission diagnoses	0.63 [0.29–1.38]	0.250	1.14 [0.49–2.67]	0.761
Time from ICU admission to HA-BSI				
Acquired prior to ICU admission	Ref.		Ref.	
Early ICU-acquired (≤7 days)	2.76 [1.25–6.07]	0.011	1.04 [0.49–2.21]	0.918
Late ICU-acquired (>7 days)	0.67 [0.31–1.42]	0.307	0.58 [0.28–1.22]	0.150
Ventilation status				
Invasive mechanical ventilation	Ref.		Ref.	
Non-invasive mechanical ventilation or CPAP	0.49 [0.23–1.05]	0.066	0.63 [0.26–1.52]	0.303
High flow oxygen nasal canula	0.60 [0.31–1.18]	0.139	0.79 [0.35–1.78]	0.565
Low flow oxygen or no oxygen	0.42 [0.23–0.76]	0.004	0.77 [0.37–1.63]	0.499
Source control				
Not required	Ref.		Ref.	
Required, achieved	0.68 [0.45–1.01]	0.058	0.46 [0.28–0.77]	0.003
Required, but NOT achieved	2.59 [1.67–4.02]	<0.001	2.02 [1.15–3.54]	0.015
Gram-positive monobacterial HA-BSIs (*n* = 93)	Univariate analysis		Multivariate analysis	
Variable	HR [95% CI]	*P* Value	aHR [95% CI]	*P* Value
SOFA score	1.28 [1.21–1.37]	<0.001	1.29 [1.17–1.43]	<0.001
COVID-19 admission diagnosis	2.00 [1.12–3.58]	0.019	1.41 [0.77–2.57]	0.265
Appropriate therapy	0.55 [0.29–1.26]	0.065	0.44 [0.17–1.11]	0.083
Antimicrobial resistance	1.44 [0.81–2.57]	0.099	0.74 [0.36–1.52]	0.414
Age	1.05 [1.03–1.06]	<0.001	1.05 [1.03–1.08]	<0.001
Source control				
Not required	Ref.		Ref.	
Required, achieved	0.70 [0.35–1.41]	0.317	0.41 [0.20–0.87]	0.019
Required, but NOT achieved	3.84 [1.70–8.66]	0.001	2.29 [0.82–6.41]	0.116

In the model built for monobacterial Gram-negative HA-BSIs, polymicrobial infections (*n* = 37) and infections caused by *Stenotrophomonas maltophilia* (*n* = 18) were excluded. In Gram-positive monobacterial HA-BSI model, only polymicrobial infections were excluded. In this model, ‘antimicrobial resistance’ indicates the presence of methicillin resistance among coagulase-negative *staphylococci* spp., and *Staphylococcus aureus*, as well as the presence of vancomycin resistance in *Enterococci* spp.

aHR, adjusted hazard ratio; HA-BSI, hospital-acquired bloodstream infection; 24/7, 24 hours a day, 7 days a week; NYHA, New York Heart Association; TDM, therapeutic drug monitoring; CPAP, continuous positive airway pressure.

## Discussion

This large prospective study including at least one teaching and/or non-teaching hospital from each geographical region of Turkey provides contemporary data of clinical and microbiological epidemiology of HA-BSIs treated in Turkish ICUs. Antimicrobial resistance rates were very high overall, and carbapenem resistance was especially frequent among GNB.

Antimicrobial resistance is a growing public health threat across the world, particularly in low- and middle-income countries.^17,18^ In this study, carbapenem resistance was very common in *Klebsiella* spp., *Pseudomonas* spp. and *Acinetobacter* spp., with a significant association with longer time to appropriate therapy and higher rate of mortality. Conversely, appropriate therapy had no significant association with survival. In a prospective multicentre Turkish cohort study evaluating BSIs caused by carbapenem-resistant *Klebsiella pneumoniae* (CRKP), appropriate therapy did not have a protective effect on mortality.^19^ Similar to our study, most patients were treated with tigecycline- and colistin-based regimens in this study.

Considering inadequate pharmacokinetic/pharmacodynamic indexes, limited clinical efficacy and increased risk of toxicity, the CLSI removed the susceptibility category of polymyxins, suggesting unforeseeable clinical efficacy of colistin for GNB infections.^20^ Consistently, 40% of our patients treated with colistin-containing regimens died at 28-day follow-up, despite the causative microorganisms being susceptible to colistin *in vitro*. Furthermore, there is an increasing tendency in colistin resistance rate among carbapenem-resistant GNB due to its widespread use in Turkey. Isler *et al*. recently reported that colistin resistance rate was 77% in CRKP isolates in Turkey.^19^ Likewise, Aslan *et al*. showed 50% colistin resistance rate among CRKP isolates collected between 2014 and 2019 in a Turkish university hospital.^21^ Although contemporary clinical guidelines and guidance documents recommend the use of new generation beta-lactam/beta-lactamase inhibitors for the treatment of severe infections caused by carbapenem-resistant *Enterobacterales* and drug-resistant *P. aeruginosa*, ceftazidime-avibactam was used in only one patient and there was no patient treated with ceftolozane-tazobactam in our study.^22,23^ This finding is mostly related to the fact that these antibiotics were not yet within the scope of reimbursement in Turkey at the time of patient enrolment in this study.

In a subgroup analysis of monobacterial Gram-negative HA-BSIs treated with appropriate therapy, early appropriate therapy was not found significantly associated with survival. This finding may be subsequent to several confounding factors and should be evaluated cautiously. Even though a similar finding was observed in the EUROBACT II study, interpretation of the interrelationship between early appropriate therapy and survival is very difficult in observational studies.^8^ In a large-scale retrospective cohort study from Sweden, delays in appropriate antimicrobial treatment significantly ramped up the risk of 30-day mortality after 12 hours from blood culture collection (aOR 1.17, 95% CI 1.01–1.37). Patients with the onset of BSI in the ICU were not included in this study, and 85.3% of the episodes were community-onset. Furthermore, the most common causative pathogens were *E. coli*, *S. aureus* and viridans *streptococci*, while very few episodes of BSI occurred with antimicrobial-resistant pathogens (4.0%). Septic shock was present in only 5.7% of episodes, which was considerably lower than in our cohort. In a subgroup analysis including only BSI of antimicrobial-resistant pathogens (*n* = 420), the risk of mortality in inappropriate therapy group was higher at all time points. Despite its important findings, this study’s results do not represent our patient populations’ epidemiology and characteristics.^24^ In another retrospective cohort study including community-onset BSIs, Lee *et al*. showed that appropriate antimicrobial therapy should be administered within the first 48 hours after non-critically ill patients arrive at the emergency department. However, in severely ill patients (Pitt bacteraemia score ≥4), appropriate therapy should be empirically given within 1 hour of patients’ arrival at the emergency department.^25^ In parallel with these studies, a systematic review and meta-analysis reported that the mortality risk was significantly lower with molecular rapid diagnostic methods than with standard microbiology methods (OR 0.73, 95% CI 0.54–0.80), particularly in the presence of antimicrobial stewardship programme.^26^ On the other hand, in a multicentre randomized controlled trial, implementation of a rapid diagnostic method ensured timely antibiotic escalations to occur almost 2 days faster for those with antimicrobial-resistant Gram-negative bacteraemia.^27^ Despite this benefit, rapid diagnostic method-based approach was shown to not provide any survival benefit over standard of care. In this trial, only five patients infected with carbapenem-resistant GNB were included and appropriate empirical antimicrobial therapy rate was very high in the standard of care arm due to low rate of antimicrobial resistance. Therefore, these results cannot be extrapolated to our settings and further randomized clinical trials exploring the impact of early appropriate therapy on the clinical outcomes of carbapenem-resistant GNB infections are highly warranted.

The last report of Turkish national antimicrobial resistance surveillance system, computed from 2011 data, showed that 54.7% of *S. aureus* and 42.9% of *Enterococcus faecium* isolated in blood culture samples of ICU patients had methicillin resistance and vancomycin resistance, respectively.^28^ The lower frequency of antimicrobial-resistant Gram-positive bacterial isolates in our study compared to national surveillance data is parallel with decreasing trend in frequency of MRSA in HA-BSIs in Europe and many other regions.^8,29^ The rate of methicillin resistance among coagulase-negative *Staphylococcus* species was very high in our study (78.1%), in line with the results of the EUROBACT II study (73.3%). Although these pathogens were not evaluated in Turkish national antimicrobial resistance surveillance study, a study published in 2007 from a Turkish university hospital reported that methicillin resistance rate is 67.5% among coagulase-negative *Staphylococcus* spp. (*n* = 200).^30^ Unlike GNB infections, antimicrobial resistance was not found as a risk factor of mortality in Gram-positive BSIs. This finding underlines the importance of the availability of highly effective antibiotics for the treatment of antimicrobial-resistant Gram-positive BSIs. Although no significant association could be demonstrated between appropriate therapy and survival in monobacterial Gram-positive HA-BSIs (aHR 0.44, 95% CI 0.17–1.11, *P* = 0.083), this result is most probably due to inadequate sample size in this group.

Several studies suggested that COVID-19 pandemic changed the epidemiology of HA-BSIs in critically ill patients. *Enterococci* were more frequently isolated in HA-BSI of COVID-19 patients, ranging from 25% to 50% of cases.^31–33^ Among the centres included in the EUROBACT II study, ICUs admitted both COVID-19 and non-COVID-19 patients (including 12 Turkish ICUs) were evaluated in a separate analysis. In this study, a significant association between COVID-19 status and mortality were shown.^31^ Similarly, in our study, *Enterococci* were the most common Gram-positive pathogens and COVID-19 status was an independent risk factor of mortality for Gram-negative HA-BSIs.

As compared with the EUROBACT II study, in this study, carbapenem resistance rates were higher in GNB isolates other than *E. coli*, where carbapenem resistance was more common in EUROBACT II patients. In the EUROBACT II study, at least one adequate antimicrobial therapy was administered more frequently within 24 hours (51.5% versus 42.6%). This is probably due to the higher rate of antimicrobial resistance in GNB isolates as well as more common methicillin resistance in *S. aureus* and coagulase-negative *Staphylococcus* isolates identified in HA-BSIs of critically ill Turkish patients. Furthermore, source control was effectively achieved in 81.8% and 73.7% of the EUROBACT II patients and Turkish patients, respectively. As a consequence of higher antimicrobial resistance rate, lower rate of source control, frequent utilization of colistin-based regimens and higher ratio of patients with COVID-19 admission diagnosis (26.0% versus 12.9%), the all-cause mortality rate was higher among Turkish patients than the entire EUROBACT II cohort (49.1% versus 37.1%). In both studies, lack of source control, higher severity of illness, COVID-19 admission diagnosis, carbapenem resistance and unavailability of clinical pharmacist consultation were found to be independent predictors of mortality. By contrast, achieving source control was an independent protective factor. Similarly, early appropriate therapy did not provide significant mortality benefit over late appropriate therapy in both studies.

The results of this study have several clinical implications. Considering the insignificant impact of appropriate therapy on survival in Gram-negative HA-BSIs, limitations of colistin-containing therapies and high colistin resistance rates among carbapenem-resistant GNB isolates in Turkey, novel beta-lactam/beta-lactamase inhibitors should be prioritized for the treatment of carbapenem-resistant GNB infections. Second, therapeutic drug monitoring for aminoglycosides, vancomycin and β-lactams, local infection treatment guidelines and rapid diagnostic tests for identification of resistance mechanisms were infrequently used in Turkish ICUs. Last, source control and availability of clinical pharmacists were associated with lower risk of death in Gram-negative HA-BSIs. Considering these findings and the importance of adequate antimicrobial exposure at the site of infection in critically ill patients, a multidisciplinary management approach should be adopted in Turkish ICUs. Furthermore, the very high antimicrobial resistance rate indicates the need to improve infection prevention and control measures along with antimicrobial stewardship practices in Turkish ICUs.

This study is not exempt from some important limitations. First, data collection was initiated before and continued during the first year of the COVID-19 pandemic. This presumably affected the patient characteristics, types of identified microorganism, rates of antimicrobial resistance and risk factors of mortality. Although the specific data were not collected in that respect and thus not available to analyse, several factors may have contributed to this effect including but not limited to overcrowding in the ICUs leading a breach in infection control practices, use of immunosuppressive therapies including steroids and immune-modifying agents such as tocilizumab. Second, pathogen identification and antimicrobial susceptibility testing were not performed in a central laboratory by using reference methods. Last, sample size was limited in multivariable analysis of monobacterial Gram-positive HA-BSIs.

In conclusion, our study shows high 28-day mortality, particularly in patients with severe presentation, carbapenem-resistant GNB bacteraemia and COVID-19 admission diagnosis. However, source control and availability of clinical pharmacists were associated with survival. Therefore, a multifaceted management programme is required for optimal treatment of Turkish ICU patients with HA-BSI.

## Supplementary Material

dkad167_Supplementary_Data

## Supplementary data

Figure S1 and Tables S1 to S10 are available as Supplementary data at *JAC* Online.
