# Supplementary material for: Epidemiology and risk factors of 28-day mortality of hospital-acquired bloodstream infection in Turkish intensive care units: a prospective observational cohort study
Source: J Antimicrob Chemother. 2023 Jun 2;78(7):1757–68. doi: 10.1093/jac/dkad167 (PMC10376926; doi:10.1093/jac/dkad167)
Supplement: dkad167_Supplementary_Data [file dkad167_supplementary_data.docx]

**Supplementary Table S1. Characteristics of participating ICUs according to survival status at day-28**

| **Characteristics** | **All ICUs**  **(n=24)** | **All patients (N= 547)** | **Non-survivors  (n= 269)** | **Survivors  (n= 278)** | **P value** |
| --- | --- | --- | --- | --- | --- |
| **Academic status of the hospital** | | | | | <.001 |
| Teaching hospital | 22 (91.7) | 500 (91.4) | 259 (96.3) | 241 (86.7) |  |
| Non-teaching hospital | 2 (8.3) | 47 (8.6) | 10 (3.7) | 37 (13.3) |  |
| **Funding of the hospital** | | | | | 0.833 |
| Public | 20 (83.3) | 456 (83.4) | 221 (82.2) | 235 (84.5) |  |
| Private | 4 (16.7) | 91 (16.6) | 48 (17.6) | 43 (15.5) |  |
| **Type of ICU** | | | | | 0.008 |
| Mixed (medical-surgical) | 20 (83.3) | 404 (73.9) | 185 (68.8) | 219 (78.8) |  |
| Medical | 4 (16.7) | 143 (26.1) | 84 (31.2) | 59 (21.2) |  |
| **Structure of the ICU** | | | | | 0.178 |
| Closed-ICU | 19 (79.2) | 440 (80.4) | 207 (77.0) | 233 (83.8) |  |
| Open-ICU | 5 (20.8) | 107 (19.6) | 62 (23.0) | 45 (16.2) |  |
| **Specific Recruitment** | | | | | |
| General ICU | 24 (100.0) | 547 (100.0) | 269 (100.0) | 278 (100.0) | NC |
| Paediatric | 3 (12.5) | 49 (9.0) | 14 (5.2) | 35 (12.6) | 0.173 |
| Cardiac-surgical | 4 (16.7) | 86 (15.7) | 44 (16.4) | 42(15.1) | 0.528 |
| Coronary-care | 3 (12.5) | 59 (10.8) | 23 (8.6) | 36 (12.9) | 0.779 |
| Post-operative | 17 (70.8) | 404 (73.9) | 194 (72.1) | 210 (75.5) | 0.545 |
| Neuro-surgical | 16 (66.7) | 386 (70.6) | 178 (66.2) | 208 (74.8) | 0.207 |
| Trauma | 16 (66.7) | 377 (68.9) | 173 (64.3) | 204 (73.4) | 0.193 |
| Burns | 2 (8.3) | 28 (5.1) | 11 (4.1) | 17 (6.1) | 0.798 |
| **Number of ventilator equivalent beds in the ICU** < **15** | 13 (54.2) | 305 (55.7) | 124 (46.1) | 181 (65.1) | 0.084 |
| **Nurse to ventilator-bed ratio** | 2.2 [1.7-2.5] | 2.3 [1.8-2.6] | 2.3 [1.8-2.6] | 2.3 [1.8-3.0] | 0.196 |
| **Senior doctor to ventilator-bed ratio** | 7 [5.4-10.1] | 7 [5.3-11.0] | 7 [5.3-11.0] | 7 [5.3-11.0] | 0.961 |
| **Senior medical cover is available 24/7** | 19 (79.2) | 435 (79.5) | 222 (82.5) | 213 (76.6) | 0.087 |
| **General surgery is available 24/7** | 24 (100.0) | 547 (100.0) | 269 (100.0) | 278 (100.0) | NC |
| **Infectious diseases specialist or clinical microbiologist are consulted** | | | | | 0.393 |
| Available when requested 24/7 | 20 (87.0) | 457 (85.1) | 222 (83.8) | 235 (86.4) |  |
| As a permanent staff of the ICU | 3 (13.0) | 80 (14.9) | 43 (16.2) | 37 (13.6) |  |
| **Clinical pharmacists are consulted** | | | | | 0.038 |
| Available when requested 24/7 | 3 (13.0) | 58 (11.2) | 21 (8.1) | 37 (14.3) |  |
| Never or sporadically | 20 (87.0) | 459 (88.8) | 237 (91.9) | 222 (85.7) |  |
| **TDM of aminoglycosides is available** | | | | | 0.279 |
| Everyday | 1 (4.2) | 10 (1.8) | 3 (1.1) | 7 (2.5) |  |
| At least once a week | 3 (12.5) | 45 (8.2) | 19 (7.1) | 26 (9.4) |  |
| Not available | 20 (80.3) | 492 (89.9) | 247 (91.8) | 245 (88.1) |  |
| **TDM of vancomycin is available** | | | | | 0.049 |
| Everyday | 2 (8.3) | 37 (6.8) | 24 (8.9) | 13 (4.7) |  |
| At least once a week | 4 (16.7) | 52 (9.5) | 20 (7.4) | 32 (11.5) |  |
| Not available | 18 (75.0) | 458 (83.7) | 225 (83.6) | 233 (83.8) |  |
| **TDM of β-lactams is available** | | | | | 0.065 |
| Everyday | 1 (4.2) | 10 (1.8) | 3 (1.1) | 7 (2.5) |  |
| At least once a week | 2 (8.3) | 16 (2.9) | 4 (1.5) | 12 (4.3) |  |
| Not available | 21 (87.5) | 521 (95.2) | 262 (97.4) | 259 (93.2) |  |

Results reported as n (%) for categorical variables and median [IQR] for continuous variables.

24/7, 24 hours a day, 7 days a week; ICU, intensive care unit; TDM, therapeutic drug monitoring. Ventilator equivalent beds refers to the maximum number of ventilated patients the ICU can accommodate at one time.

**Supplementary Table S2. Baseline characteristics of the patients according the mortality status at day-28**

| **Variable** | **All patients**  **(n=547)** | **Non-survivors**  **(n=269)** | **Survivors**  **(n=278)** | **P value** |
| --- | --- | --- | --- | --- |
| **Chronic illnesses** | | | | |
| Moderate COPD | 65 (11.9) | 33 (12.3) | 32 (11.5) | 0.784 |
| Severe COPD | 20 (3.7) | 7 (2.6) | 13 (4.7) | 0.287 |
| Heart failure (NYHA 3) | 59 (10.3) | 40 (14.9) | 19 (6.8) | 0.004 |
| Heart failure (NYHA 4) | 22 (4.0) | 9 (3.3) | 13 (4.7) | 0.566 |
| Previous myocardial infarction | 46 (8.4) | 27 (10.0) | 19 (6.8) | 0.232 |
| Peripheral vascular disease | 15 (2.7) | 9 (3.3) | 6 (2.2) | 0.556 |
| Cerebrovascular disease | 85 (15.5) | 28 (10.4) | 57 (20.5) | 0.001 |
| Dementia | 53 (9.7) | 28 (10.4) | 25 (9.0) | 0.576 |
| Hemiplegia | 26 (4.8) | 5 (1.9) | 21 (7.6) | 0.002 |
| Diabetes without end organ damage | 93 (17.0) | 41 (15.2) | 52 (18.7) | 0.281 |
| Diabetes with end organ damage | 60 (11.0) | 44 (16.4) | 16 (5.8) | <.001 |
| Renal disease, moderate | 32 (5.9) | 14 (5.2) | 18 (6.5) | 0.652 |
| Renal disease, severe (chronic dialysis) | 32 (5.9) | 16 (5.9) | 16 (5.8) | 0.924 |
| Connective tissue disease | 14 (2.6) | 6 (2.2.) | 8 (2.9) | 0.835 |
| Ulcer disease (gastro-duodenal) | 13 (2.4) | 3 (1.1) | 10 (3.6) | 0.089 |
| Liver disease, mild to moderate | 5 (0.9) | 1 (0.4) | 4 (1.4) | 0.373 |
| Liver disease, severe | 7 (1.3) | 5 (1.9) | 2 (0.7) | 0.279 |
| **Immunosuppression** | | | | |
| Steroids | 17 (3.1) | 10 (3.7) | 7 (2.5) | 0.574 |
| Chemotherapy/Radiotherapy within 6 months | 44 (8.0) | 21 (7.8) | 23 (8.3) | 0.965 |
| Targeted therapy for cancer | 13 (2.4) | 8 (3.0) | 5 (1.8) | 0.412 |
| Organ Transplant | 10 (1.8) | 6 (2.2.) | 4 (1.4) | 0.539 |
| AIDS | 1 (0.2) | 1 (0.4) | 0 (0.0) | 0.492 |
| Other immunosuppression | 29 (5.3) | 17 (6.3) | 12 (4.3) | 0.393 |
| **Source of ICU admission** | | | | 0.079 |
| Hospital ward/floor | 202 (36.9) | 104 (38.7) | 98 (35.3) |  |
| Emergency department | 244 (44.6) | 109 (40.5) | 135 (48.6) |  |
| Other hospital | 64 (11.7) | 41 (15.2) | 23 (8.3) |  |
| Operating Room/recovery | 23 (4.2) | 8 (3.0) | 15 (5.4) |  |
| Other intermediate care unit | 9 (1.6) | 4(1.5) | 5 (1.8) |  |
| Others | 5 (0.9) | 3 (1.1.) | 2 (0.7) |  |
| **Primary diagnosis at ICU admission** | | | | NC |
| Sepsis or septic shock | 48 (8.8) | 24 (8.9) | 24 (8.6) |  |
| Cardiac arrest | 20 (3.7) | 9 (3.3) | 11 (4.0) |  |
| Cardio-vascular causes | 23 (4.2) | 6 (2.2) | 17 (6.1) |  |
| Gastro-intestinal causes | 13 (2.4) | 7 (2.6) | 6 (2.2) |  |
| Hypovolemic or haemorrhagic shock | 4 (0.7) | 3 (1.1) | 1 (0.4) |  |
| Metabolic causes | 12 (2.2) | 5 (1.9) | 7 (2.5) |  |
| Multiple trauma (no TBI) | 17 (3.1) | 5 (1.9) | 12 (4.3) |  |
| Neurologic causes | 86 (15.7) | 22 (8.2) | 64 (23.0) |  |
| COVID-19* | 142 (26.0) | 104 (38.7) | 38 (13.7) |  |
| Post-Operative admission | 27 (4.9) | 10 (3.7) | 17 (6.1) |  |
| Renal failure | 13 (2.4) | 5(1.9) | 8 (2.9) |  |
| Respiratory admission* | 136 (24.9) | 68 (25.3) | 68 (24.5) |  |
| Traumatic brain injury | 4 (0.7) | 0 (0.0) | 4 (1.4) |  |
| Other | 2 (0.4) | 1 (0.4) | 1 (0.4) |  |

Results are presented as n(%). * Respiratory admission refers to admission for respiratory failure other than COVID-19. COPD, chronic obstructive pulmonary disease; ICU, intensive care unit; NYHA, New York heart association; AIDS, acquired immunodeficiency syndrome; TBI, Traumatic brain injury.

**Supplementary Table S3. Appropriate antimicrobial therapies according to 28-day survival status**

| **Antimicrobial** | **Alive on day-28 (n=314)** | **Death on day-28 (n=360)** | **Total (n=674)** |
| --- | --- | --- | --- |
| Amphotericin B Lipid Complex, n (%) | 1 (0.3) | 1 (0.3) | 2 (0.3) |
| Liposomal Amphotericin B, n (%) | 5 (1.6) | 6 (1.7) | 11 (1.6) |
| Amikacin, n (%) | 8 (2.5) | 9 (2.5) | 17 (2.5) |
| Amphotericin B deoxycholate, n (%) | 1 (0.3) | 0 | 1 (0.1) |
| Ampicillin-sulbactam, n (%) | 1 (0.3) | 6 (1.7) | 7 (1.0) |
| Ampicillin, n (%) | 1 (0.3) | 0 | 1 (0.1) |
| Anidulafungin, n (%) | 8 (2.5) | 8 (2.2) | 16 (2.4) |
| Caspofungin, n (%) | 1 (0.3) | 3 (0.8) | 4 (0.6) |
| Cefazolin, n (%) | 0 | 2 (0.6) | 2 (0.3) |
| Cefepime, n (%) | 3 (1.0) | 4 (1.1) | 7 (1.0) |
| Cefoperazone-sulbactam, n (%) | 5 (1.6) | 4 (1.1) | 9 (1.3) |
| Cefotaxime, n (%) | 0 | 1 (0.3) | 1 (0.1) |
| Ceftazidime-avibactam, n (%) | 0 | 1 (0.3) | 1 (0.1) |
| Ceftazidime, n (%) | 3 (1.0) | 8 (2.2) | 11 (1.6) |
| Ceftriaxone, n (%) | 3 (1.0) | 12 (3.3) | 15 (2.2) |
| Ciprofloxacin, n (%) | 2 (0.6) | 5 (1.4) | 7 (1.0) |
| Colistin, n (%) | 88 (28.0) | 56 (15.6) | 144 (21.3) |
| Daptomycin, n (%) | 3 (1.0) | 1 (0.3 | 4 (0.6) |
| Doxycycline, n (%) | 0 | 1 (0.3) | 1 (0.1) |
| Ertapenem, n (%) | 3 (1.0) | 5 (1.4) | 8 (1.2) |
| Fluconazole, n (%) | 4 (1.3) | 13 (3.6) | 17 (2.5) |
| Fosfomycin, n (%) | 0 | 2 (0.6) | 2 (0.3) |
| Gentamicin, n (%) | 4 (1.3) | 2 (0.6) | 6 (0.9) |
| Imipenem-cilastatin, n (%) | 10 (3.2) | 9 (2.5) | 19 (2.8) |
| Levofloxacin, n (%) | 5 (1.6) | 4 (1.1) | 9 (1.3) |
| Linezolid, n (%) | 8 (2.5) | 10 (2.8) | 18 (2.6) |
| Meropenem, n (%) | 38 (12.1) | 88 (24.4) | 126 (18.7) |
| Micafungin, n (%) | 4 (1.3) | 5 (1.4) | 9 (1.3) |
| Moxifloxacin, n (%) | 1 (0.3) | 0 | 1 (0.1) |
| Piperacillin-tazobactam, n (%) | 18 (5.7) | 34 (9.4) | 52 (7.7) |
| Polymyxin B, n (%) | 9 (2.9) | 4 (1.1) | 13 (2.0) |
| Rifampicin, n (%) | 0 | 2 (0.6) | 2 (0.3) |
| Ceftriaxone, n (%) | 1 (0.3) | 0 | 1 (0.1) |
| Trimethoprim sulfamethoxazole, n (%) | 7 (2.2) | 7 (1.9) | 14 (2.1) |
| Teicoplanin, n (%) | 39 (12.4) | 26 (7.2) | 65 (9.6) |
| Tigecycline, n (%) | 17 (5.4) | 9 (2.5) | 26 (3.9) |
| Vancomycin, n (%) | 13 (4.1) | 12 (3.3) | 25 (3.7) |

**Supplementary Table S4. Characteristics of participating ICUs in monobacterial Gram-negative HA-BSIs according to survival status at day-28**

| **Characteristics** | **All patients (N= 329)** | **Non-survivors  (n= 158)** | **Survivors  (n= 171)** | **P value** |
| --- | --- | --- | --- | --- |
| **Academic status of the hospital** | | | | <.001 |
| Teaching hospital | 302 (91.8) | 154 (97.5) | 148 (86.5) |  |
| Non-teaching hospital | 27 (8.2) | 4 (2.5) | 23 (13.5) |  |
| **Type of ICU** | | | | 0.087 |
| Mixed (medical-surgical) | 257 (78.1) | 117 (74.1) | 140 (81.9) |  |
| Medical | 72 (21.9) | 41 (25.9) | 31 (18.1) |  |
| **Number of ventilator equivalent beds in the ICU** ≥ **15** | 150 (45.6) | 61 (52.0) | 89 (38.6) | 0.014 |
| **Senior medical cover is available 24/7** | 265 (80.5) | 134 (84.8) | 131 (76.6) | 0.082 |
| **Infectious diseases specialist or clinical microbiologist are consulted** | | | | 0.144 |
| Available when requested 24/7 | 270 (83.1) | 125 (79.6) | 145 (86.3) |  |
| As a permanent staff of the ICU | 55 (16.9) | 32 (20.4) | 23 (13.7) |  |
| **Clinical pharmacists are consulted** | | | | 0.026 |
| Available when requested 24/7 | 34 (11.0) | 10 (6.6) | 24 (15.2) |  |
| Never or sporadically | 275 (89.0) | 141 (93.4) | 134 (84.8) |  |
| **TDM of aminoglycosides is available** | | | | 0.266 |
| Everyday | 9 (2.7) | 2 (1.3) | 7 (4.1) |  |
| At least once a week | 31 (9.4) | 14 (8.9) | 17 (9.9) |  |
| Not available | 289 (87.8) | 142 (89.9) | 147 (86.0) |  |
| **TDM of vancomycin is available** | | | | 0.620 |
| Everyday | 24 (7.3) | 13 (8.2) | 11 (6.4) |  |
| At least once a week | 36 (10.9) | 15 (9.5) | 21 (12.3) |  |
| Not available | 269 (81.8) | 130 (82.3) | 139 (81.3) |  |
| **TDM of β-lactams is available** | | | | 0.088 |
| Everyday | 9 (2.7) | 2 (1.3) | 7 (4.1) |  |
| At least once a week | 14 (4.3) | 4 (2.5) | 10 (5.8) |  |
| Not available | 306 (93.0) | 152 (96.2) | 154 (90.1) |  |

Results reported as n (%). 24/7, 24 hours a day, 7 days a week; ICU, intensive care unit; TDM, therapeutic drug monitoring. Ventilator equivalent beds refers to the maximum number of ventilated patients the ICU can accommodate at one time.

**Supplementary Table S5. Baseline characteristics of patients with monobacterial Gram-negative HA-BSIs according the mortality status at day-28**

| **Variable** | **All patients**  **(n=329)** | **Non-survivors (n=158)** | **Survivors (n=171)** | **P value** |
| --- | --- | --- | --- | --- |
| **Patient characteristics on ICU admission** | | | | |
| **Age (years)** | 68 [56-77] | 70 [59-78] | 66 [53-77] | 0.022 |
| **SAPS II score** | 49 [38-61] | 49 [40-66] | 48 [37-59] | 0.123 |
| **Male gender** | 194 (59.0) | 99 (62.7) | 95 (55.6) | 0.191 |
| **Body Mass Index (kg/ m^2^)** | | | | 0.439 |
| <18.5 | 8 (2.4) | 3 (1.9) | 5 (2.9) |  |
| [18.5-30[ | 248 (75.4) | 124 (78.5) | 124 (72.5) |  |
| ≥30 | 73 (22.2) | 31 (19.6) | 42 (24.6) |  |
| **Charlson comorbidity index** | | | | 0.026 |
| 0 | 66 (20.1) | 23 (14.6) | 43 (25.1) |  |
| 1-2 | 144 (43.8) | 79 (50.0) | 65 (38.0) |  |
| >2 | 119 (36.2) | 56 (35.4) | 63 (36.8) |  |
| **Type of ICU Admission** | | | | 0.090 |
| Medical | 291 (88.4) | 146 (92.4) | 145 (84.8) |  |
| Surgical elective | 14 (4.3) | 5 (3.2) | 9 (5.3) |  |
| Surgical emergency | 24 (7.3) | 7 (4.4) | 17 (9.9) |  |
| **Primary ICU admission diagnosis** | | | | <0.001 |
| Sepsis or septic shock | 28 (8.5) | 15 (9.5) | 13 (7.6) |  |
| Respiratory admission * | 86 (26.1) | 43 (27.2) | 43 (25.1) |  |
| COVID-19 * | 75 (22.8) | 57 (36.1) | 18 (10.5) |  |
| Post-operative admission | 14 (4.3) | 3 (1.9) | 11 (6.4) |  |
| Other admission diagnoses | 126 (38.3) | 40 (25.3) | 86 (50.3) |  |
| **Patient characteristics at HA-BSI diagnosis** | | | | |
| **Time from ICU admission to HA-BSI** | | | | 0.020 |
| Late ICU-acquired (>7 days) | 217 (66.0) | 96 (60.8) | 121 (70.8) |  |
| Acquired prior to ICU admission | 42 (12.8) | 18 (11.4) | 24 (14.0) |  |
| Early ICU-acquired (≤7 days) | 70 (21.2) | 44 (27.8) | 26 (15.2) |  |
| **Maximum temperature** | | | | 0.008 |
| <38.2°C | 229 (69.6) | 121 (76.6) | 108 (63.2) |  |
| ≥38.2°C | 100 (30.4) | 37 (23.4) | 63 (36.8) |  |
| **SOFA score** | 8 [6-11] | 9 [6-13] | 7 [5-9] | <.001 |
| **Ventilation status** | | | | 0.003 |
| Low flow oxygen or no oxygen | 43 (13.1) | 12 (7.6) | 31 (18.1) |  |
| High flow oxygen nasal canula | 24 (7.3) | 9 (5.7) | 15 (8.8) |  |
| Non-invasive mechanical ventilation or CPAP | 22 (6.7) | 7 (4.4) | 15 (8.8) |  |
| Invasive mechanical ventilation | 240 (72.9) | 130 (82.3) | 110 (64.3) |  |
| **Vasopressors (adrenaline or noradrenaline)** | 125 (38.0) | 78 (49.4) | 47 (27.5) | <.001 |
| **Carbapenem-resistant Gram-negative** | 188 (57.1) | 116 (73.4) | 72 (42.1) | <.001 |
| **Source of HA-BSI** | | | | 0.539 |
| Intravascular catheter | 92 (28.0) | 48 (30.4) | 44 (25.7) |  |
| Intra-abdominal | 14 (4.3) | 5 (3.2) | 9 (5.3) |  |
| Primary | 51 (15.5) | 22 (13.9) | 29 (17.0) |  |
| Respiratory | 126 (38.3) | 65 (41.1) | 61 (35.7) |  |
| Urinary | 33 (10.0) | 13 (8.2) | 20 (11.7) |  |
| Other | 13 (4.0) | 5 (3.2) | 8 (4.7) |  |
| **More than 1 possible source of infection** | 53 (16.1) | 21 (13.3) | 32 (18.7) | 0.181 |
| **Appropriate therapy** | 235 (71.4) | 105 (66.5) | 130 (76.0) | 0.055 |
| **Time to in vitro active antimicrobial therapy** | | | | 0.001 |
| ≤24 hours, n (%) | 140 (42.6) | 68 (43.0) | 72 (42.1) |  |
| ]24-48] hours, n (%) | 47 (14.3) | 23 (14.6) | 24 (14.0) |  |
| ]48-120] hours, n (%) | 48 (14.6) | 14 (8.9) | 34 (19.9) |  |
| > 120 hours, n (%) | 21 (6.4) | 6 (3.8) | 15 (8.8) |  |
| Never, n (%) | 73 (22.2) | 47 (29.7) | 26 (15.2) |  |
| **Source control** | | | | <.001 |
| Not required | 206 (62.6) | 100 (63.3) | 106 (62.0) |  |
| Required, achieved | 89 (27.1) | 32 (20.3) | 57 (33.3) |  |
| Required, but NOT achieved | 34 (10.3) | 26 (16.5) | 8 (4.7) |  |
| **Chronic illnesses** | | | | |
| Solid tumor, no metastasis | 27 (8.2) | 15 (9.5) | 12 (7.0) | 0.538 |
| Solid tumor, with metastasis | 31 (9.4) | 15 (9.5) | 16 (9.4) | 1.000 |
| Haematological malignancy | 21 (6.4) | 14 (8.9) | 7 (4.1) | 0.123 |
| Moderate COPD | 40 (12.2) | 19 (12.0) | 21 (12.3) | 0.944 |
| Severe COPD | 12 (3.6) | 6 (3.8) | 6 (3.5) | 1.000 |
| Heart failure (NYHA 3) | 34 (10.3) | 23 (14.6) | 11 (6.4) | 0.025 |
| Heart failure (NYHA 4) | 13 (4.0) | 4 (2.5) | 9 (5.3) | 0.262 |
| Previous myocardial infarction | 35 (10.6) | 22 (13.9) | 13 (7.6) | 0.093 |
| Peripheral vascular disease | 10 (3.0) | 6 (3.8) | 4 (2.3) | 0.529 |
| Cerebrovascular disease | 48 (14.6) | 13 (8.2) | 35 (20.5) | 0.003 |
| Dementia | 29 (8.8) | 12 (7.6) | 17 (9.9) | 0.579 |
| Hemiplegia | 17 (5.2) | 3 (1.9) | 14 (8.2) | 0.012 |
| Diabetes without end organ damage | 51 (15.5) | 20 (12.7) | 31 (18.1) | 0.171 |
| Diabetes with end organ damage | 37 (11.2) | 28 (17.7) | 9 (5.3) | 0.001 |
| Renal disease, moderate | 24 (7.3) | 11 (7.0) | 13 (7.6) | 0.991 |
| Renal disease, severe (chronic dialysis) | 15 (4.6) | 4 (2.5) | 11 (6.4) | 0.114 |
| Connective tissue disease | 10 (3.0) | 4 (2.5) | 6 (3.5) | 0.752 |
| Ulcer disease (gastro-duodenal) | 11 (3.3) | 3 (1.9) | 8 (4.7) | 0.223 |
| Liver disease, mild to moderate | 2 (0.6) | 0 (0.0) | 2 (1.2) | 0.499 |
| Liver disease, severe | 3 (0.9) | 2 (1.3) | 1 (0.6) | 0.610 |
| **Immunosuppression** | | | | |
| Steroids | 12 (3.6) | 6 (3.8) | 6 (3.5) | 1.000 |
| Chemotherapy/Radiotherapy within 6 months | 21 (6.4) | 10 (6.3) | 11 (6.4) | 1.000 |
| Targeted therapy for cancer | 9 (2.7) | 4 (2.5) | 5 (2.9) | 1.000 |
| Organ Transplant | 6 (1.8) | 3 (1.9) | 3 (1.8) | 1.000 |
| AIDS | 1 (0.3) | 1 (0.6) | 0 (0.0) | 0.480 |
| Other immunosuppression | 20 (6.1) | 11 (7.0) | 9 (5.3) | 0.679 |

Continuous variables are presented as median [IQR]. Categorical variables are presented as n (%). Closed brackets indicate inclusive of the end of the range and open brackets indicate the exclusion of the end of the range. *Respiratory admission refers to admission for respiratory failure other than COVID-19. ICU, Intensive care unit; SAPS II, Simplified Acute Physiology Score II; HA-BSI, hospital-acquired blood stream infection; CPAP, continuous positive airway pressure; COPD, chronic obstructive pulmonary disease; NYHA, New York heart association; AIDS, acquired immunodeficiency syndrome.

**Supplementary Table S6. Characteristics of participating ICUs according to survival status at day-28 in monobacterial Gram-positive HA-BSIs**

| **Characteristics** | **All patients (N= 93)** | **Non-survivors  (n= 50)** | **Survivors  (n= 43)** | **P value** |
| --- | --- | --- | --- | --- |
| **Academic status of the hospital** | | | | 0.040 |
| Teaching hospital | 83 (89.2) | 48 (96.0) | 35 (81.4) |  |
| Non-teaching hospital | 10 (10.8) | 2 (4.0) | 8 (18.6) |  |
| **Type of ICU** | | | | 0.268 |
| Mixed (medical-surgical) | 56 (60.2) | 27 (54.0) | 29 (67.4) |  |
| Medical | 37 (39.8) | 23 (46.0) | 14 (32.6) |  |
| **Number of ventilator equivalent beds in the ICU** ≥ **15** | 33 (35.5) | 17 (34.0) | 16 (37.2) | 0.747 |
| **Senior medical cover is available 24/7** | 65 (69.9) | 36 (72.0) | 29 (67.4) | 0.633 |
| **Infectious diseases specialist or clinical microbiologist are consulted** | | | | 0.176 |
| Available when requested 24/7 | 84 (94.4) | 47 (97.9) | 37 (90.2) |  |
| As a permanent staff of the ICU | 5 (5.6) | 1 (2.1) | 4 (9.8) |  |
| **Clinical pharmacists are consulted** | | | | 1.000 |
| Available when requested 24/7 | 10 (11.6) | 5 (10.9) | 5 (12.5) |  |
| Never or sporadically | 76 (88.4) | 41 (89.1) | 35 (87.5) |  |
| **TDM of aminoglycosides is available** | | | | 0.418 |
| Everyday | 1 (1.1) | 1 (2.0) | 0 (0.0) |  |
| At least once a week | 8 (8.6)) | 3 (6.0) | 5 (11.6) |  |
| Not available | 84 (90.3) | 46 (92.0) | 38 (88.4) |  |
| **TDM of vancomycin is available** | | | | 0.531 |
| Everyday | 6 (6.5) | 4 (8.0) | 2 (4.7) |  |
| At least once a week | 8 (8.6) | 3 (6.0) | 5 (11.6) |  |
| Not available | 79 (84.9) | 43 (86.0) | 36 (83.7) |  |
| **TDM of β-lactams is available** | | | | 0.364 |
| Everyday | 1 (1.1) | 1 (2.0) | 0 (0.0) |  |
| At least once a week | 1 (1.1) | 0 (0.0) | 1 (2.3) |  |
| Not available | 91 (97.8) | 49 (98.0) | 42 (97.7) |  |

Results reported as n (%). 24/7, 24 hours a day, 7 days a week; ICU, intensive care unit; TDM, therapeutic drug monitoring. Ventilator equivalent beds refers to the maximum number of ventilated patients the ICU can accommodate at one time.

**Supplementary Table S7. Baseline characteristics of patients with monobacterial Gram-positive HA-BSIs according the mortality status at day-28**

| **Variable** | **All patients**  **(n=93)** | **Non-survivors (n=50)** | **Survivors (n=43)** | **P value** |
| --- | --- | --- | --- | --- |
| **Patient characteristics on ICU admission** | | | | |
| **Age (years)** | 73 [61-82] | 78 [68-83] | 65 [48-76] | 0.001 |
| **SAPS II score** | 50 [37-58] | 50 [39-59] | 49 [32-58] | 0.135 |
| **Male gender** | 52 (55.9) | 28 (56.0) | 24 (55.8) | 0.986 |
| **Body Mass Index (kg/ m^2^)** | | | | 0.481 |
| <18.5 | 0 (0.0) | 0 (0.0) | 0 (0.0) |  |
| [18.5-30[ | 67 (72.0) | 34 (68.0) | 33 (76.7) |  |
| ≥30 | 26 (28.0) | 16 (32.0) | 10 (23.3) |  |
| **Charlson comorbidity index** | | | | 0.182 |
| 0 | 15 (16.1) | 5 (10.0) | 10 (23.3) |  |
| 1-2 | 44 (47.3) | 24 (48.0) | 20 (46.5) |  |
| >2 | 34 (36.6) | 21 (42.0) | 13 (30.2) |  |
| **Type of ICU Admission** | | | | 0.061 |
| Medical | 81 (87.1) | 47 (94.0) | 34 (79.1) |  |
| Surgical elective | 3 (3.2) | 0 (0.0) | 3 (7.0) |  |
| Surgical emergency | 9 (9.7) | 3 (6.0) | 6 (14.0) |  |
| **Primary ICU admission diagnosis** | | | | 0.227 |
| Sepsis or septic shock | 11 (11.8) | 6 (12.0) | 5 (11.6) |  |
| Respiratory admission * | 18 (19.4) | 8 (16.0) | 10 (23.3) |  |
| COVID-19 * | 35 (37.6) | 24 (48.0) | 11 (25.6) |  |
| Post-operative admission | 4 (4.3) | 2 (4.0) | 2 (4.7) |  |
| Other admission diagnoses | 25 (26.9) | 10 (20.0) | 15 (34.9) |  |
| **Patient characteristics at HA-BSI diagnosis** | | | | |
| **Time from ICU admission to HA-BSI** | | | | 0.250 |
| Late ICU-acquired (>7 days) | 48 (51.6) | 23 (46.0) | 25 (58.1) |  |
| Acquired prior to ICU admission | 13 (14.0) | 6 (12.0) | 7 (16.3) |  |
| Early ICU-acquired (≤7 days) | 32 (34.4) | 21 (42.0) | 11 (25.6) |  |
| **Maximum temperature** | | | | 0.168 |
| <38.2°C | 68 (73.1) | 40 (80.0) | 28 (65.1) |  |
| ≥38.2°C | 25 (26.9) | 10 (20.0) | 15 (34.9) |  |
| **SOFA score** | 7 [5-10] | 9 [6-12] | 6 [4-8] | <.001 |
| **Ventilation status** | | | | 0.125 |
| Low flow oxygen or no oxygen | 18 (19.4) | 6 (12.0) | 12 (27.9) |  |
| High flow oxygen nasal canula | 4 (4.3) | 2 (4.0) | 2 (4.7) |  |
| Non-invasive mechanical ventilation or CPAP | 14 (15.1) | 6 (12.0) | 8 (18.6) |  |
| Invasive mechanical ventilation | 57 (61.3) | 36 (72.0) | 21 (48.8) |  |
| **Vasopressors (adrenaline or noradrenaline)** | 34 (36.6) | 24 (48.0) | 10 (23.3) | 0.024 |
| **Resistant Gram-positive** | 38 (40.9) | 23 (46.0) | 15 (34.9) | 0.381 |
| **Source of HA-BSI** | | | | 0.489 |
| Intravascular catheter | 31 (33.3) | 18 (36.0) | 13 (30.2) |  |
| Intra-abdominal | 5 (5.4) | 2 (4.0) | 3 (7.0) |  |
| Primary | 30 (32.3) | 18 (36.0) | 12 (27.9) |  |
| Respiratory | 17 (18.3) | 9 (18.0) | 8 (18.6) |  |
| Urinary | 4 (4.3) | 2 (4.0) | 2 (4.7) |  |
| Other | 6 (6.5) | 1 (2.0) | 5 (11.6) |  |
| **More than 1 possible source of infection** | 18 (19.4) | 8 (16.0) | 10 (23.3) | 0.535 |
| **Appropriate therapy** | 83 (89.2) | 43 (86.0) | 40 (93.0) | 0.331 |
| **Time to in vitro active antimicrobial therapy** | | | | 0.597 |
| ≤24 hours, n (%) | 48 (51.6) | 25 (50.0) | 23 (53.5) |  |
| ]24-48] hours, n (%) | 16 (17.2) | 8 (16.0) | 8 (18.6) |  |
| ]48-120] hours, n (%) | 19 (20.4) | 10 (20.0) | 9 (20.9) |  |
| > 120 hours, n (%) | 3 (3.2) | 3 (6.0) | 0 (0.0) |  |
| Never, n (%) | 7 (7.5) | 4 (8.0) | 3 (7.0) |  |
| **Source control** | | | | 0.044 |
| Not required | 55 (59.1) | 30 (60.0) | 25 (58.1) |  |
| Required, achieved | 29 (31.2) | 12 (24.0) | 17 (39.5) |  |
| Required, but NOT achieved | 9 (9.7) | 8 (16.0) | 1 (2.3) |  |
| **Chronic illnesses** | | | | |
| Solid tumor, no metastasis | 6 (6.5) | 5 (10.0) | 1 (2.3) | 0.212 |
| Solid tumor, with metastasis | 7 (7.5) | 4 (8.0) | 3 (7.0) | 1.000 |
| Haematological malignancy | 2 (2.2) | 1 (2.0) | 1 (2.3) | 1.000 |
| Moderate COPD | 12 (12.9) | 6 (12.0) | 6 (14.0) | 0.779 |
| Severe COPD | 2 (2.2) | 0 (0.0) | 2 (4.7) | 0.211 |
| Heart failure (NYHA 3) | 11 (11.8) | 8 (16.0) | 3 (7.0) | 0.213 |
| Heart failure (NYHA 4) | 7 (7.5) | 3 (6.0) | 4 (9.3) | 0.700 |
| Previous myocardial infarction | 6 (6.5) | 3 (6.0) | 3 (7.0) | 1.000 |
| Peripheral vascular disease | 3 (3.2) | 2 (4.0) | 1 (2.3) | 1.000 |
| Cerebrovascular disease | 14 (15.1) | 9 (18.0) | 5 (11.6) | 0.562 |
| Dementia | 14 (15.1) | 10 (20.0) | 4 (9.3) | 0.244 |
| Hemiplegia | 3 (3.2) | 2 (4.0) | 1 (2.3) | 1.000 |
| Diabetes without end organ damage | 16 (17.2) | 8 (16.0) | 8 (18.6) | 0.740 |
| Diabetes with end organ damage | 10 (10.8) | 7 (14.0) | 3 (7.0) | 0.331 |
| Renal disease, moderate | 5 (5.4) | 2 (4.0) | 3 (7.0) | 0.659 |
| Renal disease, severe (chronic dialysis) | 10 (10.8) | 6 (12.0) | 4 (9.3) | 0.748 |
| Connective tissue disease | 4 (4.3) | 2 (4.0) | 2 (4.7) | 1.000 |
| Ulcer disease (gastro-duodenal) | 1 (1.1) | 0 (0.0) | 1 (2.3) | 0.462 |
| Liver disease, mild to moderate | 1 (1.1) | 0 (0.0) | 1 (2.3) | 0.462 |
| Liver disease, severe | 2 (2.2) | 2 (4.0) | 0 (0.0) | 0.497 |
| **Immunosuppression** | | | | |
| Steroids | 2 (2.2) | 1 (2.0) | 1 (2.3) | 1.000 |
| Chemotherapy/Radiotherapy within 6 months | 7 (7.5) | 4 (8.0) | 3 (7.0) | 1.000 |
| Targeted therapy for cancer | 1 (1.1) | 1 (2.0) | 0 (0.0) | 1.000 |
| Organ Transplant | 1 (1.1) | 1 (2.0) | 0 (0.0) | 1.000 |
| AIDS | 0 (0.0) | 0 (0.0) | 0 (0.0) | NC |
| Other immunosuppression | 3 (3.2) | 2 (4.0) | 1 (2.3) | 1.000 |

Continuous variables are presented as median [IQR]. Categorical variables are presented as n (%). Closed brackets indicate inclusive of the end of the range and open brackets indicate the exclusion of the end of the range. *Respiratory admission refers to admission for respiratory failure other than COVID-19. ICU, Intensive care unit; SAPS II, Simplified Acute Physiology Score II; HA-BSI, hospital-acquired blood stream infection; CPAP, continuous positive airway pressure; COPD, chronic obstructive pulmonary disease; NYHA, New York heart association; AIDS, acquired immunodeficiency syndrome.

**Supplementary Table S8. Characteristics of participating ICUs according to survival status at day-28 in non-COVID-19 monobacterial Gram-negative HA-BSIs**

| **Characteristics** | **All patients (N= 254)** | **Non-survivors  (n= 101)** | **Survivors  (n= 153)** | **P value** |
| --- | --- | --- | --- | --- |
| **Academic status of the hospital** | | | | 0.001 |
| Teaching hospital | 230 (90.6) | 99 (98.0) | 131 (85.6) |  |
| Non-teaching hospital | 24 (9.4) | 2 (2.0) | 22 (14.4) |  |
| **Type of ICU** | | | | 0.168 |
| Mixed (medical-surgical) | 209 (82.3) | 79 (78.2) | 130 (85.0) |  |
| Medical | 45 (17.7) | 22 (21.8) | 23 (15.0) |  |
| **Number of ventilator equivalent beds in the ICU** ≥ **15** | 129 (50.8) | 47 (46.5) | 82 (53.6) | 0.271 |
| **Senior medical cover is available 24/7** | 197 (77.6) | 82 (81.2) | 115 (75.2) | 0.331 |
| **Infectious diseases specialist or clinical microbiologist are consulted** | | | | 0.852 |
| Available when requested 24/7 | 215 (86.0) | 87 (87.0) | 128 (85.3) |  |
| As a permanent staff of the ICU | 35 (14.0) | 13 (13.0) | 22 (14.7) |  |
| **Clinical pharmacists are consulted** | | | | 0.001 |
| Available when requested 24/7 | 23 (9.7) | 2 (2.1) | 21 (14.9) |  |
| Never or sporadically | 213 (90.3) | 93 (97.9) | 120 (85.1) |  |
| **TDM of aminoglycosides is available** | | | | 0.448 |
| Everyday | 9 (3.5) | 2 (2.0) | 7 (4.6) |  |
| At least once a week | 26 (10.2) | 9 (8.9) | 17 (11.1) |  |
| Not available | 219 (86.2) | 90 (89.1) | 129 (84.3) |  |
| **TDM of vancomycin is available** | | | | 0.242 |
| Everyday | 24 (9.4) | 13 (12.9) | 11 (7.2) |  |
| At least once a week | 31 (12.2) | 10 (9.9) | 21 (13.7) |  |
| Not available | 199 (78.3) | 78 (77.2) | 121 (79.1) |  |
| **TDM of β-lactams is available** | | | | 0.356 |
| Everyday | 9 (3.5) | 2 (2.0) | 7 (4.6) |  |
| At least once a week | 14 (5.5) | 4 (4.0) | 10 (6.5) |  |
| Not available | 231 (90.9) | 95 (94.1) | 136 (88.9) |  |

Results reported as n (%). 24/7, 24 hours a day, 7 days a week; ICU, intensive care unit; TDM, therapeutic drug monitoring. Ventilator equivalent beds refers to the maximum number of ventilated patients the ICU can accommodate at one time.

**Supplementary Table S9. Baseline characteristics of the non-COVID-19 patients with monobacterial Gram-negative HA-BSIs according the mortality status at day-28**

| **Variable** | **All patients**  **(n=254)** | **Non-survivors (n=101)** | **Survivors (n=153)** | **P value** |
| --- | --- | --- | --- | --- |
| **Patient characteristics on ICU admission** | | | | |
| **Age (years)** | 67 [54-77] | 69 [58-77] | 65 [50-77] | 0.074 |
| **SAPS II score** | 51 [39-64] | 56 [42-68] | 49 [37-60] | 0.011 |
| **Male gender** | 147 (57.9) | 62 (61.4) | 85 (55.6) | 0.357 |
| **Body Mass Index (kg/ m^2^)** | | | | 0.980 |
| <18.5 | 8 (3.1) | 3 (3.0) | 5 (3.3) |  |
| [18.5-30[ | 187 (73.6) | 74 (73.3) | 113 (73.9) |  |
| ≥30 | 59 (23.2) | 24 (23.8) | 35 (22.9) |  |
| **Charlson comorbidity index** | | | | 0.012 |
| 0 | 48 (18.9) | 11 (10.9) | 37 (24.2) |  |
| 1-2 | 108 (42.5) | 52 (51.5) | 56 (36.6) |  |
| >2 | 98 (38.6) | 38 (37.6) | 60 (39.2) |  |
| **Type of ICU Admission** | | | | 0.274 |
| Medical | 218 (85.8) | 91 (90.1) | 127 (83.0) |  |
| Surgical elective | 13 (5.1) | 4 (4.0) | 9 (5.9) |  |
| Surgical emergency | 23 (9.1) | 6 (5.9) | 17 (11.1) |  |
| **Primary ICU admission diagnosis** | | | | 0.010 |
| Sepsis or septic shock | 28 (11.0) | 15 (14.9) | 13 (8.5) |  |
| Respiratory admission * | 86 (33.9) | 43 (42.6) | 43 (28.1) |  |
| Post-operative admission | 14 (5.5) | 3 (3.0) | 11 (7.2) |  |
| Other admission diagnoses | 126 (49.6) | 40 (39.6) | 86 (56.2) |  |
| **Patient characteristics at HA-BSI diagnosis** | | | | |
| **Time from ICU admission to HA-BSI** | | | | 0.013 |
| Late ICU-acquired (>7 days) | 165 (65.0) | 56 (55.4) | 109 (71.2) |  |
| Acquired prior to ICU admission | 40 (15.7) | 17 (16.8) | 23 (15.0) |  |
| Early ICU-acquired (≤7 days) | 49 (19.3) | 28 (27.7) | 21 (13.7) |  |
| **Maximum temperature** | | | | 0.051 |
| <38.2°C | 180 (70.9) | 79 (78.2) | 101(66.0) |  |
| ≥38.2°C | 74 (29.1) | 22 (21.8) | 52 (34.0) |  |
| **SOFA score** | 8 [5-11] | 10 [6-13] | 7 [5-9] | <.001 |
| **Ventilation status** | | | | 0.320 |
| Low flow oxygen or no oxygen | 35 (13.8) | 10 (9.9) | 25 (16.3) |  |
| High flow oxygen nasal canula | 19 (7.5) | 6 (5.9) | 13 (8.5) |  |
| Non-invasive mechanical ventilation or CPAP | 20 (7.9) | 7 (6.9) | 13 (8.5) |  |
| Invasive mechanical ventilation | 180 (70.9) | 78 (77.2) | 102 (66.7) |  |
| **Vasopressors (adrenaline or noradrenaline)** | 93 (36.6) | 52 (51.5) | 41 (26.8) | <.001 |
| **Carbapenem-resistant Gram-negative** | 136 (53.5) | 73 (72.3) | 63 (41.2) | <.001 |
| **Source of HA-BSI** | | | | 0.934 |
| Intravascular catheter | 72 (28.3) | 32 (31.7) | 40 (26.1) |  |
| Intra-abdominal | 13 (5.1) | 4 (4.0) | 9 (5.9) |  |
| Primary | 33 (13.0) | 12 (11.9) | 21 (13.7) |  |
| Respiratory | 93 (36.6) | 36 (35.6) | 57 (37.3) |  |
| Urinary | 30 (11.8) | 12 (11.9) | 18 (11.8) |  |
| Other | 13 (5.1) | 5 (5.0) | 8 (5.2) |  |
| **More than 1 possible source of infection** | 48 (18.9) | 17 (16.8) | 31 (20.3) | 0.603 |
| **Appropriate therapy** | 177 (69.7) | 58 (57.4) | 119 (77.8) | 0.001 |
| **Time to in vitro active antimicrobial therapy** | | | | <.001 |
| ≤24 hours, n (%) | 107 (42.1) | 40 (39.6) | 67 (43.8) |  |
| ]24-48] hours, n (%) | 30 (11.8) | 10 (9.9) | 20 (13.1) |  |
| ]48-120] hours, n (%) | 40 (15.7) | 8 (7.9) | 32 (20.9) |  |
| > 120 hours, n (%) | 19 (7.5) | 5 (5.0) | 14 (9.2) |  |
| Never, n (%) | 58 (22.8) | 38 (37.6) | 20 (13.1) |  |
| **Source control** | | | | <.001 |
| Not required | 151 (59.4) | 60 (59.4) | 91 (59.5) |  |
| Required, achieved | 75 (29.5) | 21 (20.8) | 54 (35.3) |  |
| Required, but NOT achieved | 28 (11.0) | 20 (19.8) | 8 (5.2) |  |
| **Chronic illnesses** | | | | |
| Solid tumor, no metastasis | 21 (8.3) | 11 (10.9) | 10 (6.5) | 0.217 |
| Solid tumor, with metastasis | 28 (11.0) | 12 (11.9) | 16 (10.5) | 0.723 |
| Haematological malignancy | 20 (7.9) | 13 (12.9) | 7 (4.6) | 0.030 |
| Moderate COPD | 34 (13.4) | 14 (13.9) | 20 (13.1) | 0.856 |
| Severe COPD | 9 (3.5) | 3 (3.0) | 6 (3.9) | 1.000 |
| Heart failure (NYHA 3) | 22 (8.7) | 13 (12.9) | 9 (5.9) | 0.053 |
| Heart failure (NYHA 4) | 11 (4.3) | 3 (3.0) | 8 (5.2) | 0.534 |
| Previous myocardial infarction | 24 (9.4) | 11 (10.9) | 13 (8.5) | 0.523 |
| Peripheral vascular disease | 5 (2.0) | 1 (1.0) | 4 (2.6) | 0.651 |
| Cerebrovascular disease | 44 (17.3) | 10 (9.9) | 34 (22.2) | 0.018 |
| Dementia | 22 (8.7) | 9 (8.9) | 13 (8.5) | 0.909 |
| Hemiplegia | 16 (6.3) | 2 (2.0) | 14 (9.2) | 0.032 |
| Diabetes without end organ damage | 37 (14.6) | 11 (10.9) | 26 (17.0) | 0.243 |
| Diabetes with end organ damage | 25 (9.8) | 17 (16.8) | 8 (5.2) | 0.005 |
| Renal disease, moderate | 18 (7.1) | 6 (5.9) | 12 (7.8) | 0.743 |
| Renal disease, severe (chronic dialysis) | 13 (5.1) | 2 (2.0) | 11 (7.2) | **0.083** |
| Connective tissue disease | 10 (3.9) | 4 (4.0) | 6 (3.9) | 1.000 |
| Ulcer disease (gastro-duodenal) | 11 (4.3) | 3 (3.0) | 8 (5.2) | 0.534 |
| Liver disease, mild to moderate | 2 (0.8) | 0 (0.0) | 2 (1.3) | 0.519 |
| Liver disease, severe | 3 (1.2) | 2 (2.0) | 1 (0.7) | 0.565 |
| **Immunosuppression** | | | | |
| Steroids | 11 (4.3) | 5 (5.0) | 6 (3.9) | 0.758 |
| Chemotherapy/Radiotherapy within 6 months | 19 (7.5) | 8 (7.9) | 11 (7.2) | 0.828 |
| Targeted therapy for cancer | 9 (3.5) | 4 (4.0) | 5 (3.3) | 0.744 |
| Organ Transplant | 6 (2.4) | 3 (3.0) | 3 (2.0) | 0.685 |
| AIDS | 1 (0.4) | 1 (1.0) | 0 (0.0) | 0.398 |
| Other immunosuppression | 17 (6.7) | 8 (7.9) | 9 (5.9) | 0.525 |

Continuous variables are presented as median [IQR]. Categorical variables are presented as n (%). Closed brackets indicate inclusive of the end of the range and open brackets indicate the exclusion of the end of the range. *Respiratory admission refers to admission for respiratory failure other than COVID-19. ICU, Intensive care unit; SAPS II, Simplified Acute Physiology Score II; HA-BSI, hospital-acquired blood stream infection; CPAP, continuous positive airway pressure; COPD, chronic obstructive pulmonary disease; NYHA, New York heart association; AIDS, acquired immunodeficiency syndrome.

**Supplementary Table S10. Multivariable cox frailty regression analysis of monobacterial Gram-negative HA-BSIs in the subgroup with non-COVID-19 admission diagnosis**

| **Variable** | **aHR [95% CI]** | ***p*-Value** |
| --- | --- | --- |
| **Clinical pharmacists are consulted** | 0.06 [0.01 – 0.41] | 0.004 |
| **Maximum temperature ≥38.2 °C** | 0.93 [0.52 – 1.66] | 0.803 |
| **SOFA score** | 1.29 [1.20 – 1.39] | <.001 |
| **Carbapenem resistance** | 3.32 [1.93 – 5.72] | <.001 |
| **Appropriate therapy** | 0.58 [0.34 – 1.01] | 0.056 |
| **Heart failure (NYHA 3)** | 1.59 [0.74 – 3.41] | 0.229 |
| **Cerebrovascular disease** | 0.57 [0.27 – 1.20] | 0.141 |
| **Haematological malignancy** | 2.46 [1.12 – 5.39] | 0.025 |
| **Diabetes with end organ damage** | 4.07 [1.92 – 8.67] | <.001 |
| **Charlson comorbidity index** | 0.89 [0.62 – 1.26] | 0.500 |
| **Primary ICU admission diagnosis** |  |  |
| Sepsis and septic shock | Ref. |  |
| Respiratory | 2.91 [1.26 – 6.71] | 0.012 |
| Post-operative admission | 0.67 [0.15 – 2.93] | 0.594 |
| Other admission diagnoses | 2.15 [0.81 – 5.68] | 0.122 |
| **Time from ICU admission to HA-BSI** |  |  |
| Acquired prior to ICU admission | Ref. |  |
| Early ICU-acquired (≤7 days) | 1.02 [0.43 – 2.44] | 0.965 |
| Late ICU-acquired (>7 days) | 0.60 [0.28 – 1.29] | 0.193 |
| **Source control** |  |  |
| Not required | Ref. |  |
| Required, achieved | 0.38 [0.19 – 0.74] | 0.004 |
| Required, but NOT achieved | 1.10 [0.54 – 2.21] | 0.797 |

In this model, patients with COVID-19 admission diagnosis, polymicrobial infections and infections caused by *Stenotrophomonas maltophilia* were excluded. ICU, intensive care unit; aHR, adjusted hazard ratio; HA-BSI, hospital-acquired bloodstream infection; NYHA, New York Heart Association; SOFA, sequential organ failure assessment.

Centre participate in study (n=28)

Patients included (n=590)

Centre excluded (n=4 11 patients)

Patients excluded (n=32)

- Community acquired (n=13)
- Missing core data (n=13)
- Contaminant (n=5)
- Retrospective inclusion (n=1)

Analysed centres (n=24)

Analysed patients (n=547)

Supplementary Figure S1. Flowchart of patient inclusion
